# Gender-specific and dose-dependent responses to L-se-methylselenocysteine are mediated by the gut microbiota-metabolite axis: implications for intestinal homeostasis and safe clinical application

**DOI:** 10.3389/fnut.2026.1803630

**Published:** 2026-03-19

**Authors:** Hui Zhang, Zhu Hong Wu, Xiao Mao Sun, Chuan Sun, Jun Chang, Lei Yuan

**Affiliations:** 1 Jiangxi Chuanqi Pharmaceutical Co., Ltd, Nanchang, Jiangxi, China; 2 School of Sports and Health, Jiangxi Science and Technology Normal University, Nanchang, Jiangxi, China; 3 Key Laboratory of Natural Microbial Medicine Research of Jiangxi Province, Jiangxi Science and Technology Normal University, Nanchang, Jiangxi, China; 4 School of Life Science, Jiangxi Science and Technology Normal University, Nanchang, Jiangxi, China

**Keywords:** gender-specific toxicity, gut microbiota-metabolite axis, intestinal health, L-SeMC, L-se-methylselenocysteine

## Abstract

**Introduction:**

L-Se-methylselenocysteine is a prominent naturally occurring organic selenium compound with notable health benefits and validated efficacy in managing various diseases. However, its impacts on intestinal microecology and the role of the gut microbiota-metabolite axis in mediating host health outcomes remain unclear.

**Methods:**

Sprague–Dawley rats were subjected to a 90-day chronic toxicity study, combined with integrated intestinal microbiome and metabolome analysis, to explore L-Se-methylselenocysteine’s effects and underlying mechanisms.

**Results:**

L-Se-methylselenocysteine at doses of 0.25–0.75 mg/kg bw/ day enhanced gut microbiota biodiversity, enriched probiotic abundance, ameliorated hematological and serum biochemical indices, and promoted synthesis of beneficial metabolites via modulating the gut microbiota-metabolite axis. Notably, high-dose L-Se-methylselenocysteine (2.25 mg/kg bw/day) induced irreversible hepatosplenic injury in female rats but not males, with gender-specific responses mediated by the axis.

**Discussion:**

L-Se-methylselenocysteine confers intestinal health benefits through the gut microbiota-metabolite axis, while defining a safe dosage range. This study provides a solid scientific basis for the rational application of L-Se-methylselenocysteine as a selenium supplement.

## 1 Introduction

Selenium is an essential trace element in human body (1) and plays a vital role in various physiological processes. As a core component of glutathione peroxidase (2) and a potent antioxidant (3), selenium mitigates oxidative stress-induced cellular damages, retards aging, and exerts critical effects including cancer prevention (4–6), cardiovascular protection (4, 7), regulation of thyroid hormone metabolism (8), and maintenance of normal reproductive function (6).

Organic selenium, where the element is covalently bound to amino acids, exhibits distinct advantages over its inorganic counterparts, including lower toxicity, higher bioactivity, superior antioxidant effects, and enhanced immune modulation (9). Additionally, it demonstrates more efficient regulation of thyroid function, auxiliary hepatoprotection effects, and improvements of sperm quality (10). Furthermore, the absorption rate of organic selenium is 1.2–2.0 times higher than that of inorganic selenium (11, 12), making it the currently preferred form for supplementation.

The primary sources of organic selenium include selenium-enriched yeast, selenomethionine, and L-Se-methylselenocysteine (L-SeMC). Selenium-enriched yeast serves as a sustained-release reservoir for selenium in the body, but it has limitations such as complex composition and potential residues of inorganic selenium. Selenomethionine is characterized by high absorption efficiency and direct incorporation to proteins, making it suitable for addressing severe selenium deficiency. However, it also carries risks, including tissue accumulation toxicity, impaired protein function due to nonspecific replacement of methionine, and a narrow safe dosage range. In contrast, L-SeMC, which naturally found in garlic and broccoli (13), possesses unique merits. It offers a well-defined chemical structure and can be *in vivo* metabolized via the reduction of methaneseleninic acid to methylselenol. Methylselenol is a key anticancer molecule (14, 15) and thereby exhibits significant chemopreventive potential against cancer (16). Additionally, L-SeMC has demonstrated efficacy in treatment of diseases such as vulvar candidiasis (17), ferroptosis (18), neuropathology and cognitive deficits (19), and anaplastic thyroid carcinoma (20).

Notably, selenium metabolism and its biological/toxic effects are closely linked to probiotics and gut flora. Beyond their role in absorption and conversion, certain bacteria and probiotics can also actively synthesize organic Se compounds and biogenic selenium nanoparticles, which may present unique bioavailability and biological activities (21). As beneficial components of the gut microecology, probiotics can promote selenium absorption and conversion into bioavailable forms—for example, gut microbiota can metabolize Se-methylselenocysteine into selenomethionine to enhance host utilization (21). Meanwhile, appropriate selenium supplementation may selectively promote beneficial gut microbes and inhibit pathogenic bacteria, synergistically regulating gut homeostasis (21). Moreover, selenium toxicity exhibits distinct gender differences: animal experiments confirm that female and male rats show different target organ sensitivities and toxic responses to selenium compounds like selenium yeast and L-SeMC at the same dosage (22). This difference may stem from gender-related variations in gut microbiota composition, selenium-metabolizing enzyme activity, and hormone levels (21), as selenium’s sex-specific effects on inflammatory responses are closely associated with gut flora (23), forming a complex regulatory axis among selenium, probiotics/gut microbiota, and gender-specific toxicity.

**Figure 1:**
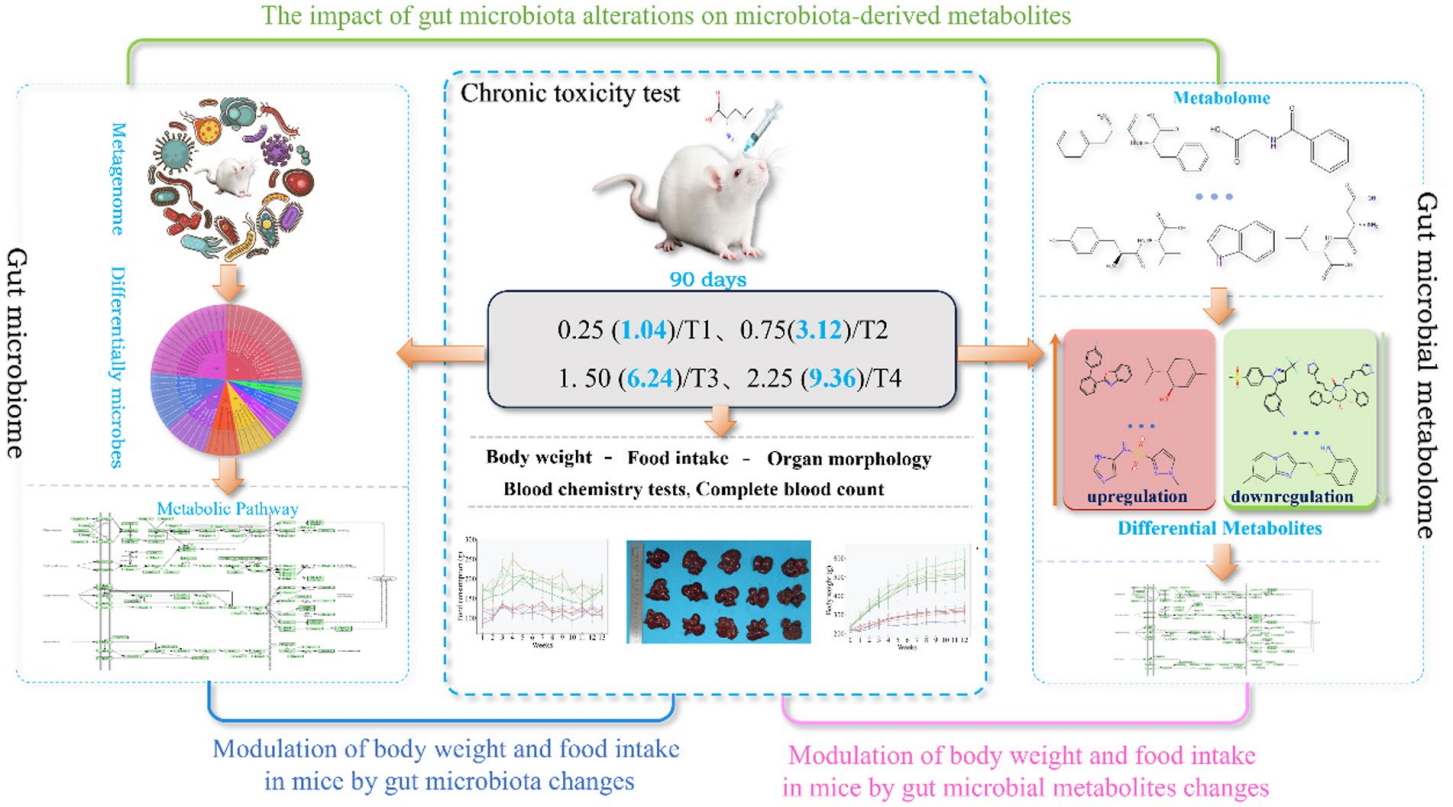
Study architecture illustrating the interrelationship between oral L-SeMC administration and alterations in the gut microbiota and metabolic profile. Numbers in parentheses indicate the human equivalent dose, converted for a 60 kg person (unit: mg/60 kg bw/day).

Intestinal microecology, defined by the collective assembly of gut microbiota and their metabolites, is closely associated with human health. As a key regulator of host physiology, it participates in multiple essential processes, including nutrient absorption, immune system maturation, intestinal barrier maintenance, and metabolic regulation. Dysbiosis, or imbalance of this microecology, is a recognized driver of various pathologies. Conversely, its restoration and homeostasis are fundamental to sustaining overall health, enhancing resistance to pathogens, and maintaining systemic metabolic balance. Studies revealed that intestinal microflora is involved in the metabolism of organic selenium compounds (24, 25), such as selenocyanate, se-methylselenocysteine, and 1β-methylseleno-N-acetyl-d-galactosamine. Several studies have explored the association between selenium, gut flora and health benefits, such as selenium-enriched probiotics regulating gut flora to improve antioxidant capacity (26) and organic selenium altering gut microbiota to alleviate metabolic disorders (27). However, the impact of L-SeMC administration on the gut flora and metabolome, as well as the mechanistic basis for its health benefits, remains unexplored, as most of these studies focus on other selenium forms rather than L-SeMC. Therefore, this study aims to evaluate the health effects of oral L-SeMC supplementation and, more importantly, to elucidate the mechanistic link between L-SeMC-induced alterations in intestinal microecology and the resultant health improvements. (Figure 1). This investigation not only clarify the intrinsic connection between L-SeMC and host health but also offers deeper insights into the mechanisms underlying its beneficial effects, thereby establishing a scientific foundation for its rational application in health maintenance.

## 2 Materials and methods

### 2.1 Test compound

L-SeMC (CAS No. 26046-90-2; purity: 99.9%) was supplied by Jiangxi Chuanqi Pharmacy Co., Ltd. (Nanchang, China). The compound was dissolved and diluted to the required test concentrations using purified water (Wahaha Group Co., Ltd., Hangzhou, China). All dosing formulations were prepared weekly based on the mean body weight of rats within the same group and stored at 4 °C.

### 2.2 Animals

Eighty Specific-pathogen-free Sprague–Dawley rats (equal numbers of males and females) were obtained from Jiangsu HuaChuangXinNuo Pharmaceutical Co., Ltd. (license number: SCXK (Su) 2025–0012). The animals were housed under controlled environmental conditions with a temperature of 20–25 °C, humidity of 40–70%, and a 12-h light/dark cycle. Standard rodent chow diet and bottled water were available *ad libitum*. After a one-week acclimatization period, the rats were randomly allocated into five groups (8 males and 8 females per group): Control (0 mg/kg bw/day), T1 (0.25 mg/kg bw/day), T2 (0.75 mg/kg bw/day), T3 (1.50 mg/kg bw/day), and T4 (2.25 mg/kg bw/day). L-SeMC was administered daily by oral gavage for 90 consecutive days.

Body weights and food intakes of the rats were recorded weekly. On the day of 90, fresh fecal samples were collected and immediately stored at −80 °C for subsequent microbiome and metabolome analyses. A forced swimming test (FST) was also conducted on the same day to assess potential effects of L-SeMC on behavioral indices of mental state. On day 91, all animals were anesthetized and euthanized. Blood samples were collected for Complete Blood Count (CBC) and Blood Biochemistry Panel (BCP) assays. Major organs (heart, liver, spleen, kidney, and lung) were dissected for gross morphological observation and histopathological examination using hematoxylin–eosin (H&E) staining.

All experimental procedures were reviewed and approved by the Institutional Animal Care and Use Committee of Jiangxi Science and Technology Normal University (3601020137931). The oral toxicity test was conducted in accordance with the Organization for Economic Co-operation and Development (OECD) guideline (28).

### 2.3 Gut microbiome and metabolome analyses

The collected fecal samples were sent to Wuhan Metware Biotechnology Co., Ltd. (Wuhan, China) for microbiome and metabolome profiling. For microbiome analysis, metagenomic sequencing was performed on the rat fecal samples. Subsequent bioinformatics processing included the annotation of taxonomic composition and functional characteristics, such as metabolic pathways, enzymes, KEGG ortholog terms (KOs), and functional modules, along with the quantification of microbial relative abundance. For metabolome analysis, an untargeted metabolomics approach was employed to characterize small-molecule metabolites. Raw data acquired via liquid chromatography-mass spectrometry (LC–MS) were processed through peak detection, alignment, and normalization. Metabolite annotation was conducted by matching the obtained spectral features against four public databases: PubChem, the Human Metabolome Database (HMDB), METLIN, and the Kyoto Encyclopedia of Genes and Genomes (KEGG).

To comprehensively evaluate the microbial biodiversity, three key alpha-diversity indices, e.g., the Shannon diversity index (*H′*), Simpson dominance index (*D*), and Pielou evenness index (*J’*) were calculated using the follows Equations 1–3:


$$H^{\prime }=-\sum_{i=1}^{S}(p_{i}\times \log(p_{i}))$$ (1)

$$D=1-\sum_{i=1}^{S}(p_{i}^{2})$$ (2)

$$J^{\prime }=\frac{H^{\prime }}{H_{\max}^{\prime }}$$ (3)

In the formulas, *S* denotes the total count of observed species within the gut microbial community, and *p_i_* refers to the relative abundance of the *i-*th species. *H’_max_* represents the theoretical maximum value of the Shannon index (*H′*), which is achieved when all species are equally abundant.

The beta diversity of the gut microbial communities was assessed using the Bray–Curtis dissimilarity coefficient, calculated as follows (Formula 4):


$$BC_{\mathrm{ij}}=1-\frac{2\sum_{k=1}^{S}\min(n_{\mathrm{ik}},n_{\mathrm{jk}})}{\sum_{k=1}^{S}n_{\mathrm{ik}}+\sum_{k=1}^{S}n_{\mathrm{jk}}}$$ (4)

Where *BC_ij_* denotes the Bray–Curtis dissimilarity coefficient between *i-*th and *j-*th microbial communities. *S* is the total number of species (or operational taxonomic units) observed across all samples. *n_ik_* and *n_jk_* refer to the relative abundances of the *k*-th species in the community *i* and community *j*, respectively.

To identify the differential microbes, linear discriminant analysis effect size (LEfSe) and significance testing based on log fold-change (LFC) calculation were performed in parallel. Microbial features meeting both a fold change (FC) threshold of ≥2.0 and an adjusted *p*-value threshold of <0.05 in both analyses were considered candidate differential microbes. The final set of differential microbes was defined as the intersection of candidates identified by both the LEfSe and LFC approaches. For the identification of differential KOs, a standard compositional data analysis (CoDA)-based *t*-test was performed separately for male and female cohorts. Within each sex, each treatment group (T1, T2, T3, and T4) was compared against its sex-matched control group. KOs with an adjusted *p*-value <0.05 were considered significantly different.

To identify the differential metabolites, the LFC was calculated for each treatment group (T1, T2, T3, and T4) relative to sex-matched control group. Metabolites meeting both of the following criteria were defined as differential metabolites: an absolute FC ≥ 2.0 and an adjusted *p*-value < 0.05.

### 2.4 Association of body weight and food intake with gut microbiota and metabolome

To elucidate potential associations among body weight, food intake, gut microbiota composition, and gut metabolome profiles, systematic correlation analyses were performed using Spearman’s rank correlation coefficient. First, pairwise Spearman’s correlation coefficients were calculated between body weight and two gut-derived data: (1) the relative abundances of intestinal microbial taxa, and (2) the concentrations of gut metabolic features. The Benjamini–Hochberg false discovery rate (FDR) procedure was applied to adjust for multiple comparisons, and relationships with an adjusted *p*-value < 0.05 were considered statistically significant.

An identical analytical pipeline was then applied to assess correlations between daily food intake and the same gut microbiome and metabolome datasets. The same significance threshold (FDR-adjusted *p* < 0.05) was used to ensure a uniform criterion across all comparisons.

### 2.5 CBC and BCP assays

CBC and BCP analyses were performed in accordance with standard laboratory protocols, covering sample preparation, reagent handling, and automated detection using commercial kits.

Kits for alanine aminotransferase (ALT), aspartate aminotransferase (AST), albumin, alkaline phosphatase (ALP), *γ*-glutamyl transferase (GGT), total bile acid (TBA), urea, creatinine, and uric acid were purchased from Shenzhen Rayto Co., Ltd. (Shenzhen, China). Kits for total bilirubin and direct bilirubin were obtained from Changchun Huili Co., Ltd. (Changchun, China).

For serum preparation, whole blood kept at 4 °C overnight, followed by centrifugation to collect the supernatant for immediate assay. Heparin-anticoagulated whole blood was centrifuged within 30 min of collection. The resulting plasma supernatant was subsequently processed under the same conditions as serum. All samples were centrifuged to remove precipitates prior to analysis. Samples showing visible hemolysis or lipemia were excluded to prevent analytical interference. All samples were analyzed within 24 h after collection to ensure the reliability of the results.

### 2.6 Forced swim test

The forced swimming test (FST) was conducted as follows: individual rats were placed in a plastic cylinder (30 × 50 cm) filled with water to a depth of 30 cm at room temperature (22 ± 1 °C). A 5-min test session was recorded using a video system. The total duration of immobility, defined as the absence of active movements (e.g., only minimal motions necessary to keep the head above water), was manually scored from the recordings. Immediately after the test, each rat was gently dried with a towel and returned to its home cage.

### 2.7 Hematoxylin–eosin (H&E) staining

For histological assessment was performed via hematoxylin–eosin (H&E) staining, which was conducted by SCI-GO Co., Ltd. (http://sci-go.com/) following their standard operating procedures. For tissue processing prior to outsourcing, freshly harvested organs were immediately fixed in 4% paraformaldehyde at room temperature for 24 h to preserve morphological integrity. The fixed samples were then kept on ice during transportation to the service provider to minimize potential ischemia-related artifacts.

## 3 Results

### 3.1 The morphological effects of oral L-SeMC administration on organs

**Figure 2:**
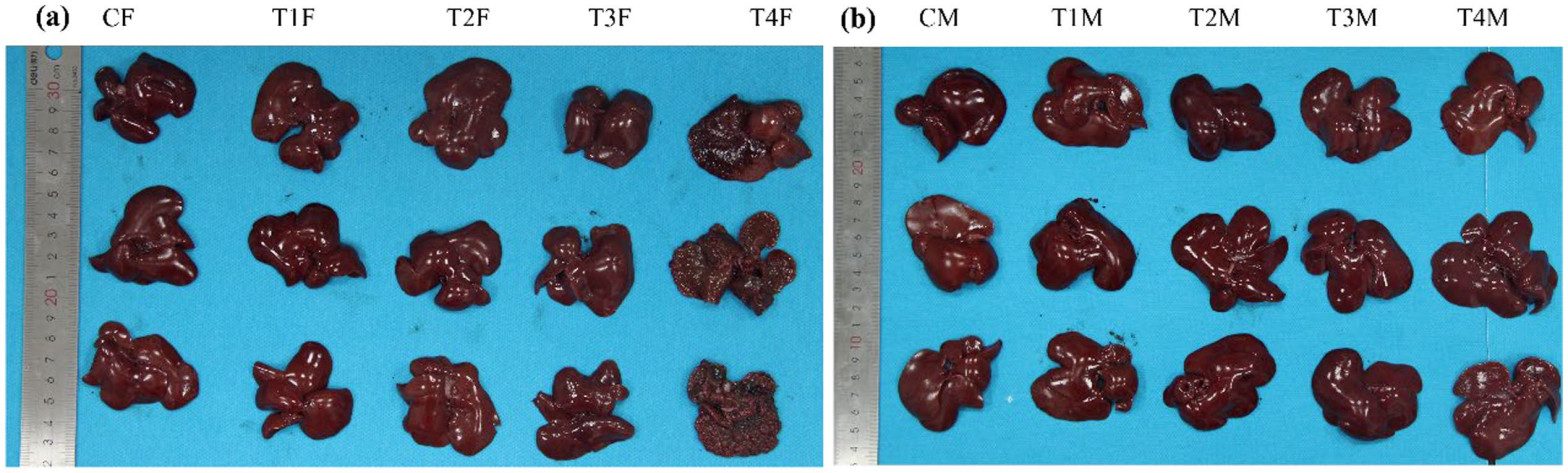
The effects of oral L-SeMC administration on the liver morphology in female **(a)** and male **(b)** rats. CF, T1F, T2F, T3F, and T4F represent the female control, female T1 (0.25 mg/kg bw/day), female T2 (0.75 mg/kg bw/day), female T3 (1.50 mg/kg bw/day), and female T4 (2.25 mg/kg bw/day) groups, respectively; CM, T1M, T2M, T3M, and T4M refer to the corresponding male treatment groups.

Morphological analyses of liver tissues (Figure 2) revealed that oral administration of L-SeMC elicited distinctly gender-divergent effects on female versus male rats, as evidenced by conspicuous disparities in macroscopic morphological phenotypes. Specifically, L-SeMC at dose of 2.25 mg/kg bw/day induced pronounced deleterious impacts on the livers of female rats. Macroscopically, these detrimental effects were characterized by overt erosive features, including superficial erosion of the liver surface, rough texture, and focal tissue defects. This female-biased susceptibility to L-SeMC-induced hepatic injury may be associated with differences in sex hormone levels or the hepatic expression profile of selenium-metabolizing enzymes. Notably, estrogen, a group of steroid hormones that play a key role in regulating the female reproductive system and secondary sexual characteristics, is well-documented to modulate drug metabolic pathways and tissue sensitivity to xenobiotics, thereby potentially exacerbating L-SeMC-associated hepatic injury in females. In sharp contrast, no statistically significant morphological alterations were observed in the livers of male rats at any dose of L-SeMC, including the highest dose. The hepatic tissues of male rats retained a normal, structurally intact surface architecture across all groups, implying that male rats may possess a more robust hepatic detoxification capacity for L-SeMC.

**Figure 3:**
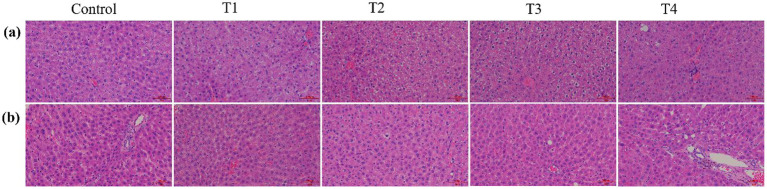
The hematoxylin–eosin staining of livers tissues from rats. **(a,b)** Representative the H&E staining images of liver sections from male and female rats, respectively. All micrographs were captured at a magnification of 20 × .

To gain mechanistic insights into the gender-biased macroscopic phenotypes and to delineate the microstructural basis of the erosive lesions observed in female rat livers, H&E staining was performed on liver tissue sections (Figure 3a). Consistent with the macroscopic observations, no significant differences were detected in the hepatic histology of male rats across all experimental groups. Specifically, the hepatic lobules retained their canonical architectural structure with clear demarcation; hepatocytes exhibited regular morphology, uniform size, and intact cell membranes. Furthermore, no characteristic signs of pathological injury, such as inflammatory cell infiltration, cytoplasmic vacuolization, or hepatocellular necrosis, were observed in the hepatic parenchyma of male rats from all treatment group. The absence of microscopic lesions in male rat livers likely attributable to the efficient clearance and detoxification of L-SeMC, whereby the coordinated action of selenium-metabolizing enzymes and endogenous antioxidant defense system may prevent the accumulation of toxic intermediates and subsequent cellular injury.

In contrast, histological examination of liver tissues from female rats in the T4 group (Figure 3b) revealed a prominent expansion of the interstitial space within the hepatic parenchyma, which corresponded to the macroscopic erosive changes. This expansion is speculated to result from L-SeMC-mediated dysregulation in the synthesis or degradation of extracellular matrix components in female rat livers. Nevertheless, consistent with the observations in male rats, no prominent pathological alterations (including inflammatory cell infiltration and fibrosis) were identified in the liver tissues of male rats. This finding suggests that the L-SeMC-induced expansion of the hepatic interstitium constitutes a female-specific morphological alteration that does not progress to a classical inflammatory response. A plausible explanation is that L-SeMC-induced perturbation is spatially confined to the interstitial compartment without directly compromising hepatocyte integrity. Alternatively, the activation of intrinsic anti-inflammatory mechanisms may effectively suppress inflammatory cascade, thereby preventing overt structural damage to hepatocytes.

**Figure 4:**
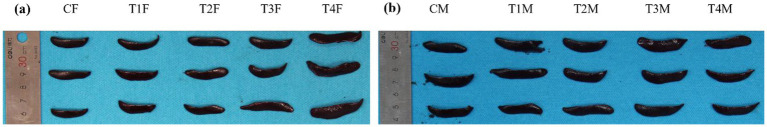
The effects of oral L-SeMC administration on the spleen morphology in female **(a)** and male **(b)** rats. CF, T1F, T2F, T3F, and T4F represent the female control, female T1 (0.25 mg/kg bw/day), female T2 (0.75 mg/kg bw/day), female T3 (1.50 mg/kg bw/day), and female T4 (2.25 mg/kg bw/day) groups, respectively; CM, T1M, T2M, T3M, and T4M refer to the corresponding male treatment groups.

The effects of different L-SeMC treatment regimens on splenic size in rats were further evaluated (Figure 4). The results revealed a striking gender-specific response pattern. For female rats (Figure 4a), splenic size in the T4 group was significantly increased relative to all other groups (*p* < 0.05), indicating a female-specific effect of high-dose L-SeMC on splenic morphology. The average spleen weights of female rats in each group were as follows: CF group 0.56 g, T1F group 0.59 g, T2F group 0.60 g, T3F group 0.56 g, and T4F group 1.11 g. The significant increase in splenic weight in the T4F group was consistent with the macroscopic enlargement observed, further confirming the female-specific effect of high-dose L-SeMC on the spleen. In contrast, no significant variations in splenic size were observed among male rats across all groups (Figure 4b). Splenic dimensions in male rats remained consistent regardless of the administered dose, suggesting that L-SeMC treatment, within the tested dosage range, did not exert a measurable regulatory effect on splenic size in males. Collectively, these data demonstrate that high-dose L-SeMC-induced splenic enlargement is a female-specific phenotypic alteration under the conditions of this study.

**Figure 5:**
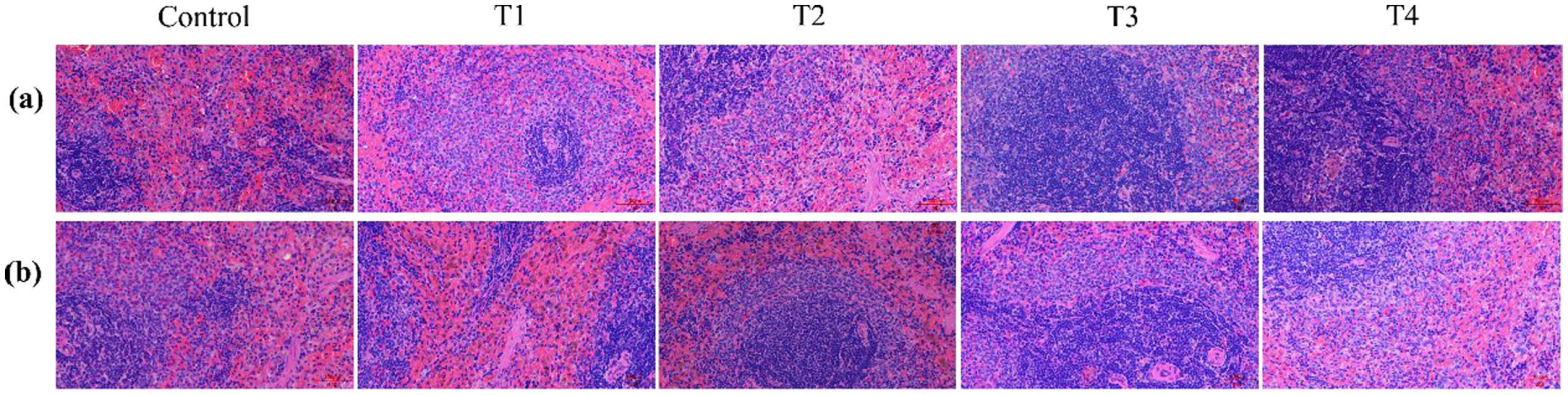
The hematoxylin–eosin staining of spleens tissues from rats. **(a,b)** represent the H&E staining images of spleen sections from male and female rats, respectively. All micrographs were captured at a magnification of 20 × .

To further determine whether the T4-mediated splenic enlargement in female rats was associated with structural injury, H&E staining was conducted to assess splenic histological integrity (Figure 5). Notably, no significant histological alterations, including inflammation, fibrosis, inflammatory cell infiltration, or other pathological lesions, were observed across all groups. Specifically, the spatial architecture of the splenic parenchyma, including the arrangement of sinusoids and cellular components, remained intact and consistent with normal histology. These findings demonstrate that the high-dose L-SeMC-induced splenic enlargement in female rats is not accompanied by overt structural damage or pathological histological changes.

Additionally, no discernible alterations in macroscopic morphology or H&E staining profiles were observed in other major organs, including the heart, lungs, and kidneys, across all groups and in both sexes. Detailed morphological data and representative H&E-stained sections for these organs are provided in Supplementary Tables 1–4 (Supplementary Figures S1–S5).

### 3.2 Biodiversity of gut microbiota

**Figure 6:**
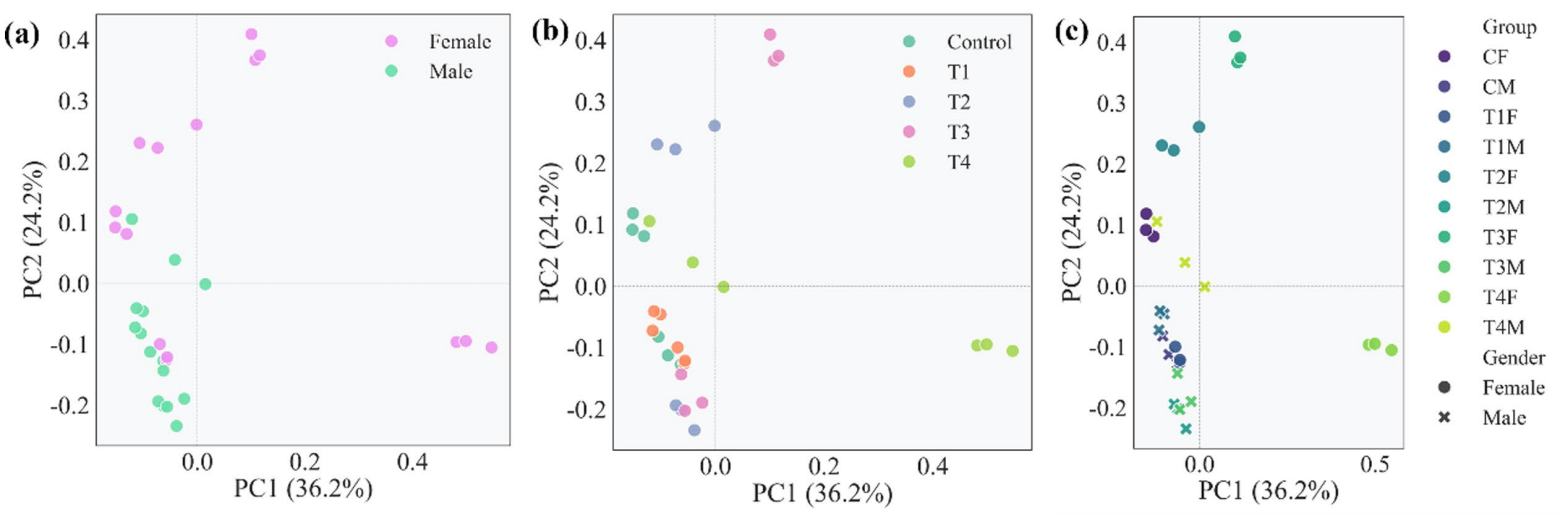
Principal component analysis of β-diversity in gut microbiome profiles. **(a)** PCA analysis of gender-driven separation of microbiome profiles; **(b)** PCA analysis of treatment-dependent clustering of microbiome profiles; **(c)** PCA analysis of interactive effects of gender and treatment on β-diversity.

Principal component analysis (PCA) was conducted to characterize the global variability across all samples, with the PC1 and PC2 collectively explaining 60.4% of the total variance (36.2 and 24.2%, respectively). As shown in Figure 6a, the PCA score plot revealed a pronounced gender-driven separation: samples from female rats were predominantly distributed along the positive axes of both PC1 (−0.2–0.5) and PC2 (−0.1–0.4), while samples from male rats clustered near the negative axes of PC1 (−0.2–0) and PC2 (−0.2–0.1). This clear segregation indicates that sex is a primary source of variability in the microbiome profiles, consistent with the gender-divergent morphological changes observed in liver and spleen tissues.

Beyond the primary gender-based stratification, the PCA also revealed treatment-related clustering patterns (Figure 6b). Samples from the control group were concentrated near the coordinate origin, whereas samples from low-to-moderate dose groups (T1-T3) exhibited a progressive shift along PC1 and PC2. Notably, samples from the high-dose group (T4) formed a distinct cluster, occupying a specific region characterized by higher PC1 (approximately −0.2 to 0.6) and lower PC2 (approximately −0.1 to 0.1) values. This pattern demonstrates that increasing doses of L-SeMC induced gradual yet discernible alterations in microbiome profiles, culminating in a state that is clearly distinct from both the control and lower-dose groups. This dose-dependent trend aligns with the macroscopic and histological evidences of hepatic injury and splenic enlargement observed specifically in female rats administered 2.25 mg/kg bw/day of L-SeMC.

When integrating both treatment group and gender into the analysis (Figure 6c), the distinct and interactive effects of these two factors became evident. Even within the same treatment group, female and male samples consistently occupied separate distinct spatial regions. For example, samples from female rats in the T4 group clustered in the upper-right quadrant, whereas samples from their male counterparts were positioned in the lower-left area. This spatial separation in the microbiome profile directly mirrors the gender-specific susceptibility to L-SeMC-induced hepatic alterations and splenic enlargement observed in the prior morphological assessments. These findings collectively demonstrate that both gender and treatment dose contribute to the variability in microbiome profiles, with gender exerting a consistent, independent effect across all treatment conditions.

**Figure 7:**
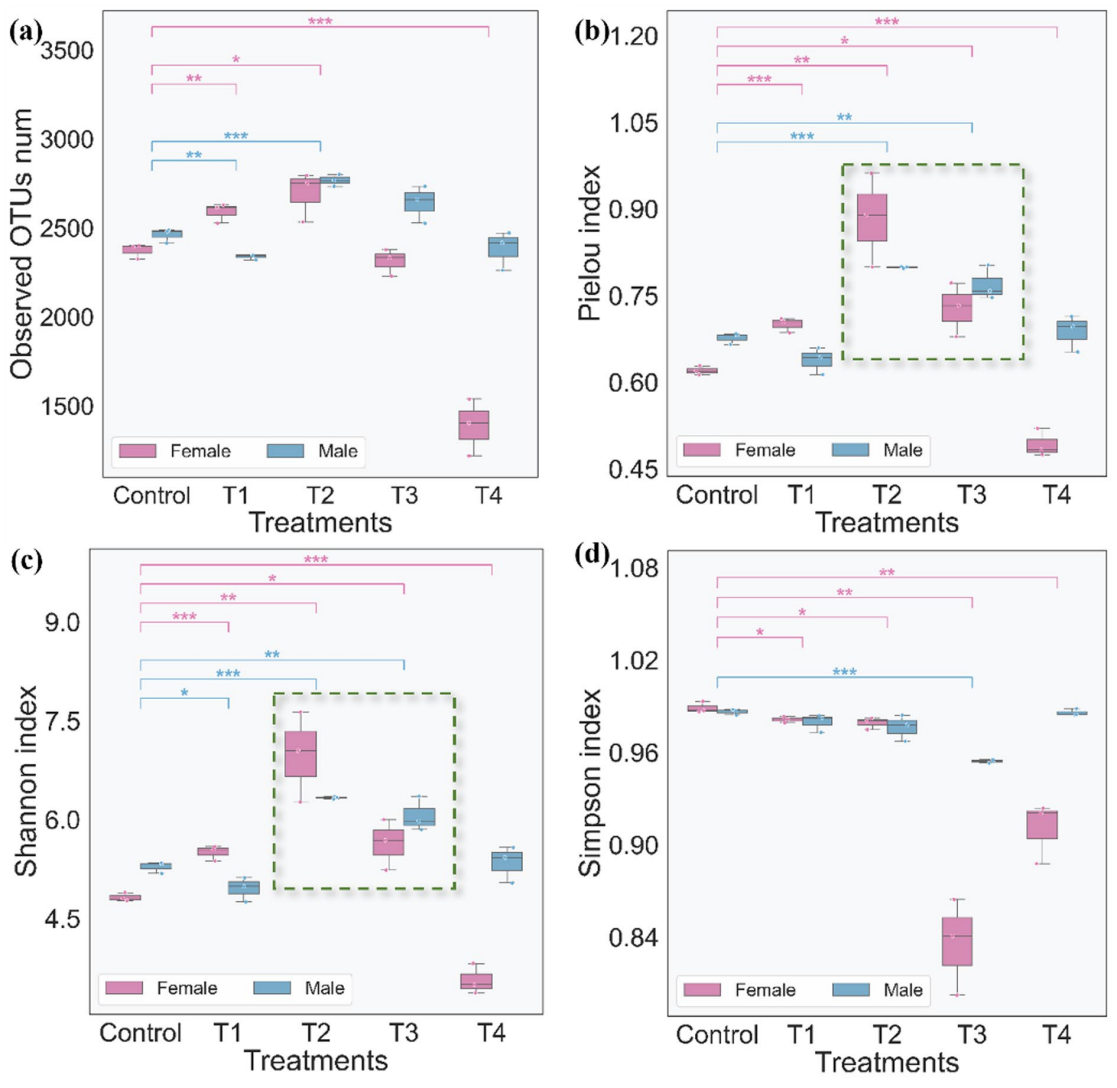
Alpha diversity analyses of rat gut microbiota. **(a)** Observed OTUs (observed operational taxonomic unit) index of rat gut microbiota; **(b)** Pielou index (microbial community evenness) of rat gut microbiota; **(c)** Shannon index (microbial richness) of rat gut microbiota; **(d)** Simpson index (microbial dominance concentration) of rat gut microbiota.

Alpha diversity analysis was performed to assess the richness and evenness of the gut microbial community across all groups, using four complementary metrics: the Observed OTUs index, Pielou evenness index, Shannon diversity index, and Simpson diversity index (Figure 7). Collectively, these indices revealed distinct gender-divergent effects of L-SeMC treatment on gut microbial diversity. Female rats exhibited far more pronounced and dose-dependent alterations in alpha diversity compared to their male counterparts.

As shown in Figure 7a, the Observed OTUs index exhibited a marked dose-dependent response in female rats. Specifically, microbial richness in the high-dose (T4) group was significantly lower (approximately 1,500 OTUs; *p* < 0.001) compared to the control and low-dose (T1-T3) groups. In stark contrast, microbial richness in male rats remained relatively stable across all groups, with no significant dose-related changes observed.

To further assess treatment-associated shifts in microbial community structure beyond richness, the Pielou evenness index was analyzed (Figure 7b). The results revealed a clear gender-biased response to L-SeMC exposure. In male rats, the Pielou index showed only minor, non-significant fluctuations in the T1 and T4 groups, but was significantly (*p* < 0.01) elevated in the T2 and T3 groups (reaching approximately 0.8 and 0.75, respectively). In female rats, the response was more dynamic, dose-dependent: the Pielou index was significantly (*p* < 0.01) increased in the T1, T2, and T3 groups (approximately 0.7, 0.9, and 0.7, respectively), but declined significantly (*p* < 0.001) in the T4 group to approximately 0.45. This dramatic decrease in community evenness in high-dose female rats paralleled the reduction in microbial richness observed in the Observed OTUs index, indicating a coordinated disruption of gut microbial community structure at the highest L-SeMC dose.

Consistently, the Shannon diversity index displayed a nearly identical trend across groups (Figure 7c). Specifically, the Shannon index was significantly (*p* < 0.01) increased in the T1, T2, and T3 groups, reflecting the combined enhancement of microbial richness and evenness in these groups. However, in female rats of the high-dose T4 group, the Shannon index exhibited a marked and statistically significant reduction (*p* < 0.001). This decrease aligned with the concurrent declines observed in both the Observed OTUs and Pielou evenness indices at the same dose. This consistency across three core alpha indices confirms that L-SeMC treatment induces synchronized, gender-specific alterations in both the compositional richness and structural evenness of the gut microbial community.

Finally, analysis of the Simpson diversity index (Figure 7d) further validated the gender-dependent impacts of L-SeMC on gut microbial diversity. In female rats, high-dose L-SeMC treatment (T3 and T4) induced a marked and significant reduction in diversity (*p* < 0.01), with index values declining to approximately 0.84 and 0.9, respectively. In contrast, microbial diversity in male rats remained stable across all treatment groups, with the only exception being a mild but statistically significant reduction (*p* < 0.001) observed in the T3 subgroup.

Taken together, these findings demonstrate that L-SeMC treatment exerts prominent gender-specific effects on gut microbial alpha diversity. Female rats exhibited greater susceptibility, showing clear dose-dependent disruptions in microbial richness, evenness, and overall community diversity. Notably, these alterations in microbial diversity parallel the gender-divergent morphological changes observed in the liver and spleen, suggesting potential crosstalk between L-SeMC-induced host tissue responses and gut microbial community dynamics.

### 3.3 Hematological and biochemical profiling of the rats

**Figure 8:**
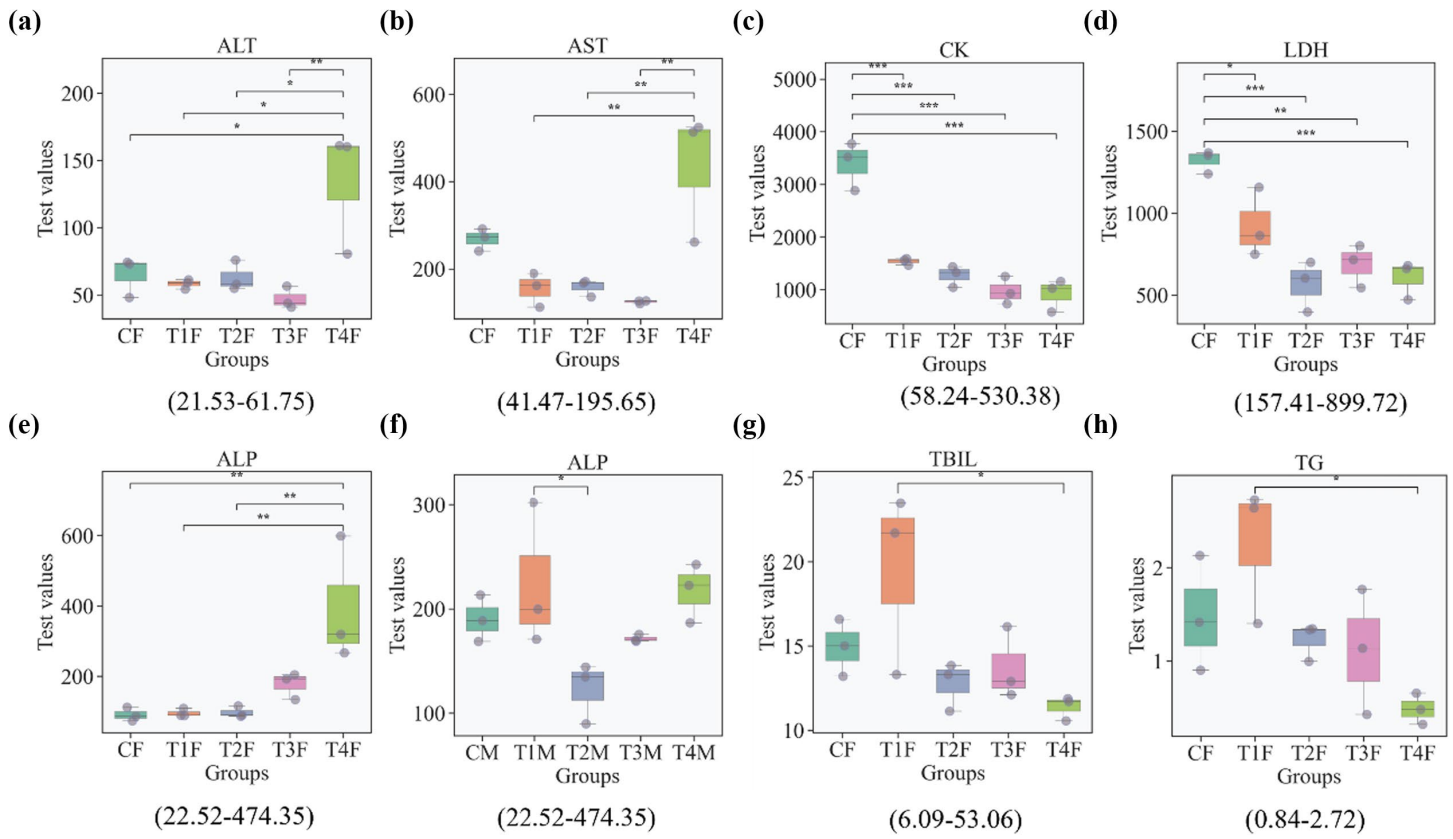
Effects of oral L-SeMC administration on serum biochemical parameters in rats. Only groups exhibiting statistically significant differences in the described indices are shown. The numbers in the parentheses below each panel indicate the corresponding normal physiological range for the specific marker. The reference ranges from laboratory-established rat normal values; analyses were performed on Chemray 240/420/800 automatic biochemical analyzers (Rayto, Shenzhen, China), calibrated with kit-matching standard reagents, and quality control was maintained throughout detection to ensure reliability; kits were purchased from Rayto (Shenzhen) and Huili (Changchun), stored at 2–8 °C, and prepared per instructions.

**Figure 9:**
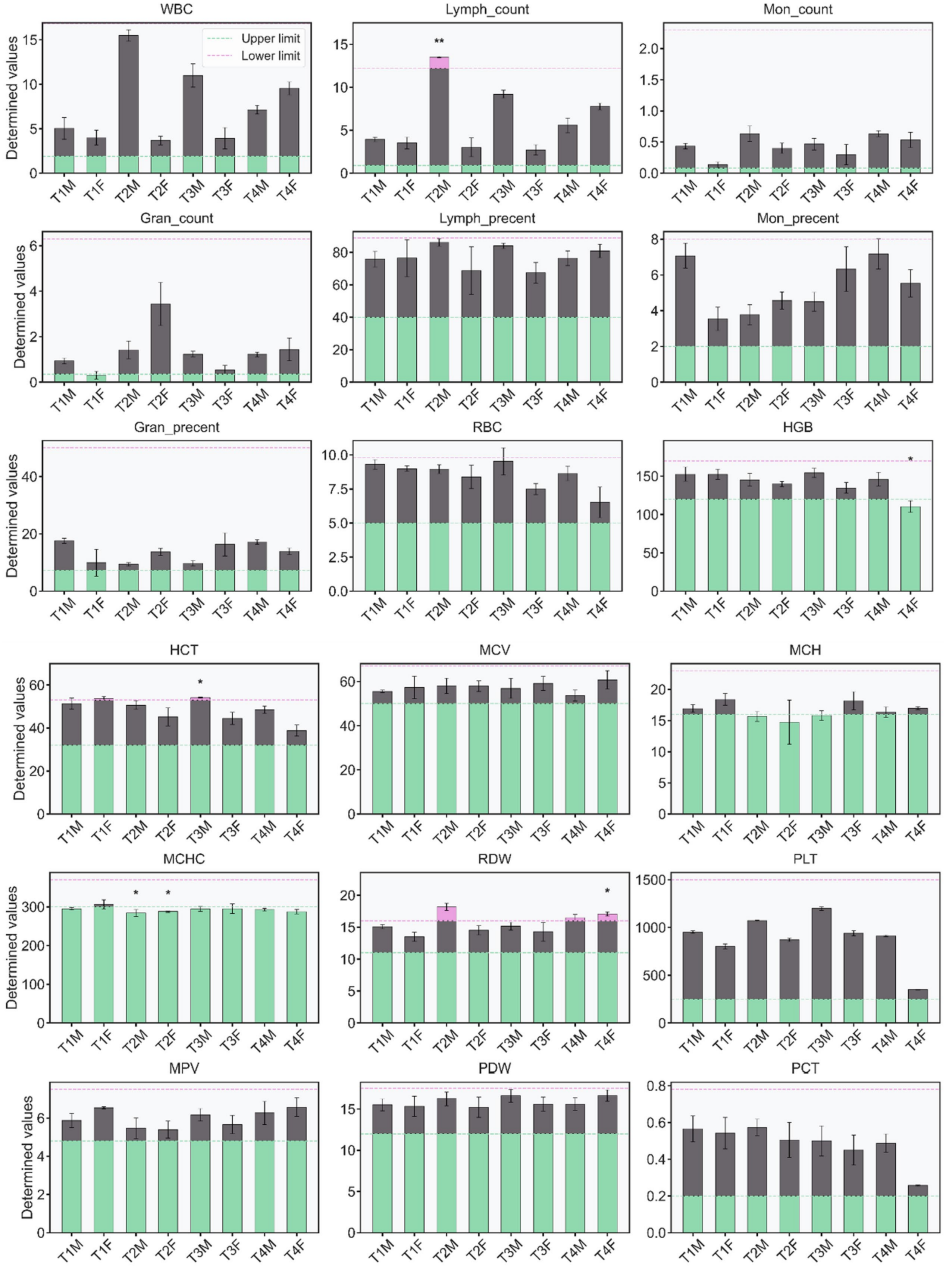
The effects of oral L-SeMC administration on the CBC markers of rats.

To comprehensively characterize the gender-divergent physiological effects of L-SeMC, serum biochemical (Figure 8) and CBC (Figure 9) analyses were performed across all experimental groups. The results substantiate and extend the prior observations of female-biased hepatic injury and gut microbial dysbiosis in high L-SeMC dose.

#### 3.3.1 Serum biochemical marker analysis

Alanine aminotransferase (ALT, a marker of hepatocellular injury) levels in control female rats were marginally elevated but remained within the normal physiological range (Figure 8a). Low-to-moderate L-SeMC doses (T1-T3) effectively downregulated the ALT concentrations. In contrast, high-dose treatment (T4) induced a significant elevation in females, with values exceeding the upper limit of the normal range. Conversely, male rats maintained stable, normal ALT levels across all treatment groups, reflecting their inherent resilience to L-SeMC-induced hepatic stress.

Consistently, aspartate aminotransferase (AST) exhibited a similar gender- and dose-dependent response (Figure 8b). AST levels in control female rats were significantly elevated above the normal range, indicating pre-existing hepatic stress in the untreated females. Low-to-moderate L-SeMC doses (T1-T3) robustly reduced AST concentrations to within normal limits, whereas the females in T4 group showed a dramatic and significant elevation of AST. Conversely, male rats exhibited no significant intergroup differences, with AST levels remaining consistently within the normal range across all groups, aligning with their intact hepatic morphology observed in prior histological analyses.

Creatine kinase (CK) and lactate dehydrogenase (LDH), two markers of systemic cellular injury, displayed analogous trends (Figures 8c,d). The levels of the both markers in control female rats exceeded their respective normal ranges. L-SeMC administration across all doses (T1-T4) significantly downregulated the both markers, though only LDH was restored to the normal physiological range.

Alkaline phosphatase (ALP, a biliary function marker) exhibited gender-specific and dose-dependent regulation, with all measured values remaining within the normal physiological range for both sexes. In female rats (Figure 8e), ALP levels in the T1 and T2 were comparable to those in the control, showing no significant differences. By contrast, ALP activity was significantly elevated in the T3 and T4 groups relative to controls, with the most pronounced increase observed in the T4 group, suggesting a subtle, dose-related perturbation of biliary homeostasis. In male rats (Figure 8f), ALP levels showed only modest fluctuations across all treatment groups, with only the T2 group exhibiting a significant but within-range reduction.

Regarding markers of bile metabolism and excretion, total bilirubin (TBIL) and triglyceride (TG) levels remained within their respective normal physiological ranges across all groups, regardless of sex or L-SeMC dose (Figures 8f–h). In female rats, TBIL and TG levels in the T1 group were significantly higher than those in T4 group, whereas no significant differences were observed between T1 and other groups. In male rats, both markers showed minimal fluctuations with no significant intergroup differences. These findings indicate that L-SeMC administration, within the tested dosage range, does not induce substantial disruption of core lipid metabolism or bile excretion pathways.

#### 3.3.2 CBC analysis

Among all assessed hematological parameters, only three, namely lymphocyte count, red cell distribution width (RDW), and mean corpuscular hemoglobin concentration (MCHC), exhibited measurable deviations from the control. In some subgroups, values fell outside the normal physiological range (Figure 9). This indicates that L-SeMC exerts only mild hematological effects, which stands in contrast to its more pronounced impacts on hepatic histology and gut microbial diversity.

Lymphocyte count, a key indicator of systemic immune cell dynamics, showed a gender-specific response. No significant alterations were detected in female rats across all treatment groups. In contrast, male rats in the T2 group exhibited an elevation slightly exceeding the upper limit of the normal reference range. This observation suggests a transient, moderate-dose-dependent stimulatory effect of L-SeMC on lymphocyte proliferation specifically in males, without inducing overt immune dysregulation in other groups.

RDW showed distinct gender- and dose-dependent alterations. RDW values in male rats of the T2 and T4 groups, as well as in female rats of the T4 group, were significantly higher than those in their respective control groups, with all elevated values exceeding the normal reference range. This implies that moderate-to-high doses of L-SeMC may perturb red blood cell maturation, with effects more pronounced in high-dose females and moderate/high-dose males. This pattern aligns with the female-biased susceptibility observed in prior biochemical analyses.

MCHC, which reflecting average hemoglobin per red blood cell, was slightly below the normal physiological range across all groups, regardless of sex or L-SeMC dose. Notably, both male and female rats in the T2 group showed significantly lower MCHC levels compared to their respective controls, whereas other treatment groups exhibited mild, non-significant reductions. Importantly, all observed deviations were mild and non-anemic, indicating that L-SeMC exerts only a subtle regulation on hemoglobin synthesis rather than inducing overt hematological toxicity.

In summary, these hematological and biochemical findings confirm the dual, dose-dependent nature of L-SeMC’s effects: low-to-moderate doses promote physiological homeostasis by normalizing deviant markers (e.g., AST, CK, and LDH in females), whereas high doses induce female-biased perturbations (e.g., elevated ALT, AST, and RDW). These mild perturbations are well below established toxicological cut-offs and consistent with Park et al.’s observation (29), while the T3 dose marks a critical inflection point where beneficial effects diminish and subtle adverse changes emerge, aligning with the gender- and dose-dependent trends in anatomical analyses. The consistency of these results with prior morphological and microbial diversity data underscores the critical role of biological sex in modulating L-SeMC’s physiological impact, thereby establishing a cohesive link between hepatic injury, gut microbial dysbiosis, and systemic physiological changes in rats.

Red and green horizontal lines represent the upper and lower limits of the normal physiological range for each parameter, respectively. Asterisks (*) indicate statistically significant differences compared with the control group (*p* < 0.05). Abbreviations: WBC, white blood cell; Lymph_count, lymphocyte count; Mon_count, monocyte count; Gran_count, granulocyte count; Lymph_percent, lymphocyte percent; Mon_percent, monocyte percent; RBC, red blood cell; HGB, hemoglobin; HCT, hematocrit; MCV, mean corpuscular volume; MCH, mean corpuscular hemoglobin; MCHC, mean corpuscular hemoglobin concentration; RDW, red blood cell distribution width; PLT, platelet count; MPV, mean platelet volume; PDW, platelet distribution width; PCT, plateletcrit. CBC analyses were performed on a BC-2800vet veterinary automatic hematology analyzer (Mindray), calibrated with matching standard products, and strict QC procedures ensured detection accuracy and repeatability.

### 3.4 The differential microbes

**Figure 10:**
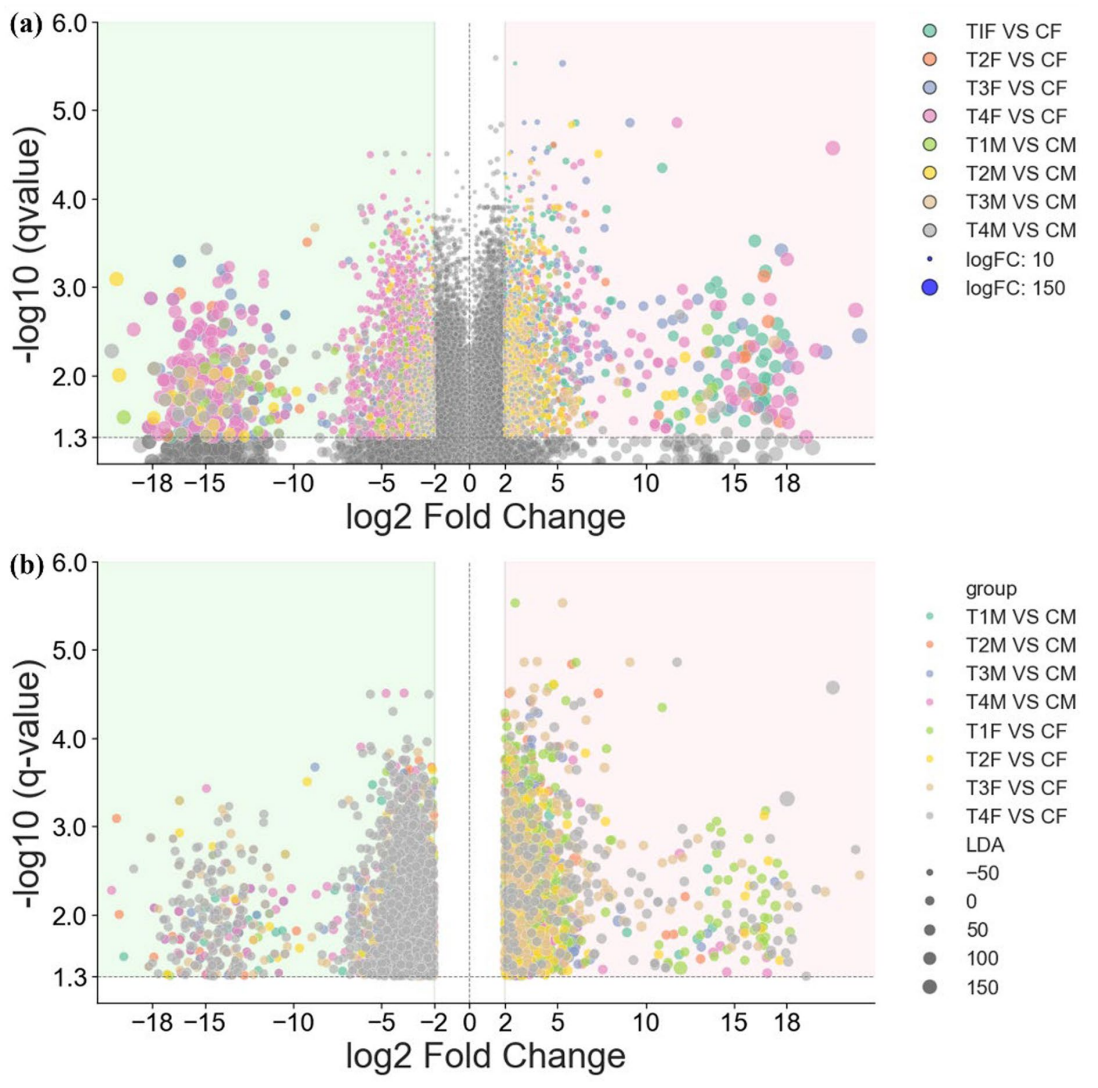
Gender-specific differential gut microbiota profiles in rats. **(a,b)** show volcano plots of differential gut microbial taxa based on log_2_ fold-change (logFC) and linear discriminant analysis (LDA) effect size, respectively. For clarity, non-differential taxa are omitted from **(b)**.

To explore potential mechanisms underlying the phenotypic differences, specifically the overt hepatic injury in T4 female rats versus the intact hepatic morphology and stable biochemical indices in T4 male rats, we analyzed gut microbial composition and functions (Figure 10, with detailed in Tables 1, 2). A distinct gender-specific microbial response to L-SeMC was observed. In T4 female rats, both logFC-based (Figure 10a) and LDA-based (Figure 10b) analyses revealed pronounced shift, characterized by a dense cluster of predominantly downregulated taxa (7 downregulated vs. 2 upregulated). In contrast, microbial alterations in male rats across all doses were modest. Specifically, in T4 male rats, all 5 differential microbes were downregulated.

**Table 1:** Microbial community analysis of gender-specific differentially microbial taxa across doses.

Group	Species	Genus	logFC	Function
T2M	*Prevotellamassilia timonensis*	*Prevotellamassilia*	6.10	Potential beneficial effect
T3F	*Bifidobacterium criceti*	*Bifidobacterium*	22.14	Beneficial effect
	*Erysipelotrichaceae bacterium* NYU BL F16	Other	20.17	Potential beneficial effect
	*Ileibacterium valens*	*Ileibacterium*	11.38	–
	*Bifidobacterium choerinum*	*Bifidobacterium*	10.94	Beneficial effect
T3M	*Pseudoscardovia radai*	*Pseudoscardovia*	8.44	Potential beneficial effect
	*Phascolarctobacterium succinatutens*	*Phascolarctobacterium*	6.56	Potential beneficial effect
	*Prevotella* sp. P3 120	*Prevotella*	−6.36	Potential beneficial effect
T4F	*Candidatus Arthromitus* sp. SFB mouse NL	*Candidatus_Arthromitus*	−15.34	Beneficial effect
	*Pseudoleptotrichia goodfellowii*	*Pseudoleptotrichia*	−15.82	–
	*Phascolarctobacterium* sp. Marseille Q4147	*Phascolarctobacterium*	−14.52	Beneficial effect
	*Bacterium* D16 56	Other	−13.82	–
	*Helicobacter labetoulli*	*Helicobacter*	−13.26	–
	*Bifidobacterium pseudolongum*	*Bifidobacterium*	12.39	Beneficial effect
	*Clostridium* sp. CAG 729	*Clostridium*	−12.38	–
	*Ruminococcus* sp. AF37 3 AC	*Ruminococcus*	−10.38	Beneficial effect
	*Turicibacter* sp. TS3	*Turicibacter*	10.16	–
	*Mammaliicoccus lentus*	*Mammaliicoccus*	10.07	–
T4M	*Prevotella* sp. P4 98	*Prevotella*	−10.09	–
	*Prevotella* sp. P5 126	*Prevotella*	−8.06	Potential beneficial effect
	*Prevotella hominis*	*Prevotella*	−7.49	Potential beneficial effect
	*Prevotella* sp. P4 119	*Prevotella*	−6.72	-
	*Bifidobacterium porcinum*	*Bifidobacterium*	−6.23	Potential beneficial effect

**Table 2:** Microbial community analysis of gender-specific differentially abundant KOs across doses.

Enriched group	Map	logFC	Function	Pathway (L1/L2/L3)
T1F	map00062	1.55	Fatty acid metabolism	Metab/Lipid/FA_elongation
	map04064	10.22	Inflammatory	EIP/ST/NFKB
T1M	map04010	−1.34	Oxidation/Inflammatory	EIP/ST/MAPK
	map04011	1.49	Oxidation/Inflammatory	EIP/ST/MAPK_yeast
	map04010	−1.34	Cancer	EIP/ST/ MAPK
T2F	map04011	−1.65	Oxidation/Inflammatory	EIP/ST/MAPK_yeast
T3F	map04011	−1.51	Oxidation/Inflammatory	EIP/ST/MAPK_yeast
T4F	map00062	2.19	Fatty acid metabolism	Metab/Lipid/FA_elongation
	map04016	1.29	Oxidation/Inflammatory	EIP/ST/MAPK_plant
	map00480	1.38	Oxidation	Metab/Metab_AA**/**GLU
	map04011	1.56	Oxidation	EIP/ST/MAPK_yeast
T4M	map04013	1.02	Oxidation	EIP/ST/MAPK_fly
	map04062	−9.74	Inflammatory	ORG/IS/CHE

Metab, Metabolism; Lipid: Lipid metabolism; FA_elongation, Fatty acid elongation; EIP, Environmental Information Processing; ST, Signal transduction; MAPK_yeast, MAPK signaling pathway-yeast; MAPK, MAPK signaling pathway; MAPK_plant, MAPK signaling pathway-plant; MAPK_fly, MAPK signaling pathway-fly; Metab_AA, Metabolism of other amino acids; GLU, Glutathione metabolism; ORG, Organismal Systems; IS, Immune system; CHE, Chemokine signaling pathway; NFKB, NF-kappa B signaling pathway.

Detailed analyses of differential gut microbiota (Table 1) and KOs (Table 2) clarify the nature of these microbial and functional shifts, enabling clear distinction between beneficial and adverse changes induced by different doses of L-SeMC. Low-to-moderate doses (T1-T3) predominantly induced beneficial or neutral microbial alterations.

In female rats of the T3 group, multiple beneficial *Bifidobacterium* species (e.g., *Bifidobacterium cricetid* and *Bifidobacterium choerinum*) were enriched, which are closely associated with gut barrier integrity and anti-inflammatory effects. In contrast, male rats in the same group showed subtle depletion of the potentially beneficial species *Phascolarctobacterium succinatutens*, without inducing overt microbial dysbiosis. Male rats in T2 group exhibited depletion of *Prevotellamassilia timonensis*, a potential beneficial species, but displayed only mild hematological perturbations and no hepatic injury, suggesting a tolerable and transient microbial response. Female rats in T1 group showed an enrichment of inflammatory pathway map04064; however, no corresponding hepatic damage was detected, likely buffered by the overall stability of the gut microbiota.

In contrast, high-dose L-SeMC induced adverse microbial and functional shifts. For instance, female rats in T4 group were depleted of multiple protective microorganisms, including *Candidatus Arthromitus* sp. SFB mouse NL, *Phascolarctobacterium* sp. Marseille Q4147, and *Ruminococcus* sp. AF37 3 AC, all of which are linked to beneficial metabolic processes or anti-inflammatory functions. Functional profiling further revealed the enrichment of pro-inflammatory signaling pathways (map04064 and map04016) and the fatty acid metabolism pathway (map00062) in this group, consistent with the elevated ALT/AST levels and hepatic injury observed in T4 female rats. Although T4 male rats also showed depletion of potentially beneficial *Prevotella* species and mild suppression of inflammatory pathways, but no hepatic injury occurred, reflecting the greater resilience of the male gut ecosystem to high-dose L-SeMC exposure.

These observed microbial and functional alterations exhibit a distinct correlation with the hematological and biochemical profiles, thereby forming an integrative mechanistic pathway. Low-to-moderate doses of L-SeMC (T1-T3) modulate the gut microbiota in a gender-specific, predominantly beneficial manner, characterized by the enrichment of beneficial taxa and accompanied by only modest physiological perturbations. In contrast, high-dose L-SeMC (T4) triggers microbial dysbiosis, particularly in females. This state is marked by the depletion of protective taxa and a functional shift toward pro-inflammatory pathways. These adverse changes are likely direct contributors to the observed elevation in hepatic injury markers, systemic cellular stress, and hematological deviations. The relative stability of the gut microbiota in males underpins their resilience to high-dose L-SeMC-induced hepatic injury. Conversely, the susceptibility of the female gut microbiota to the high-dose disruption explains their greater overall physiological vulnerability.

### 3.5 The differential gut metabolites

**Figure 11:**
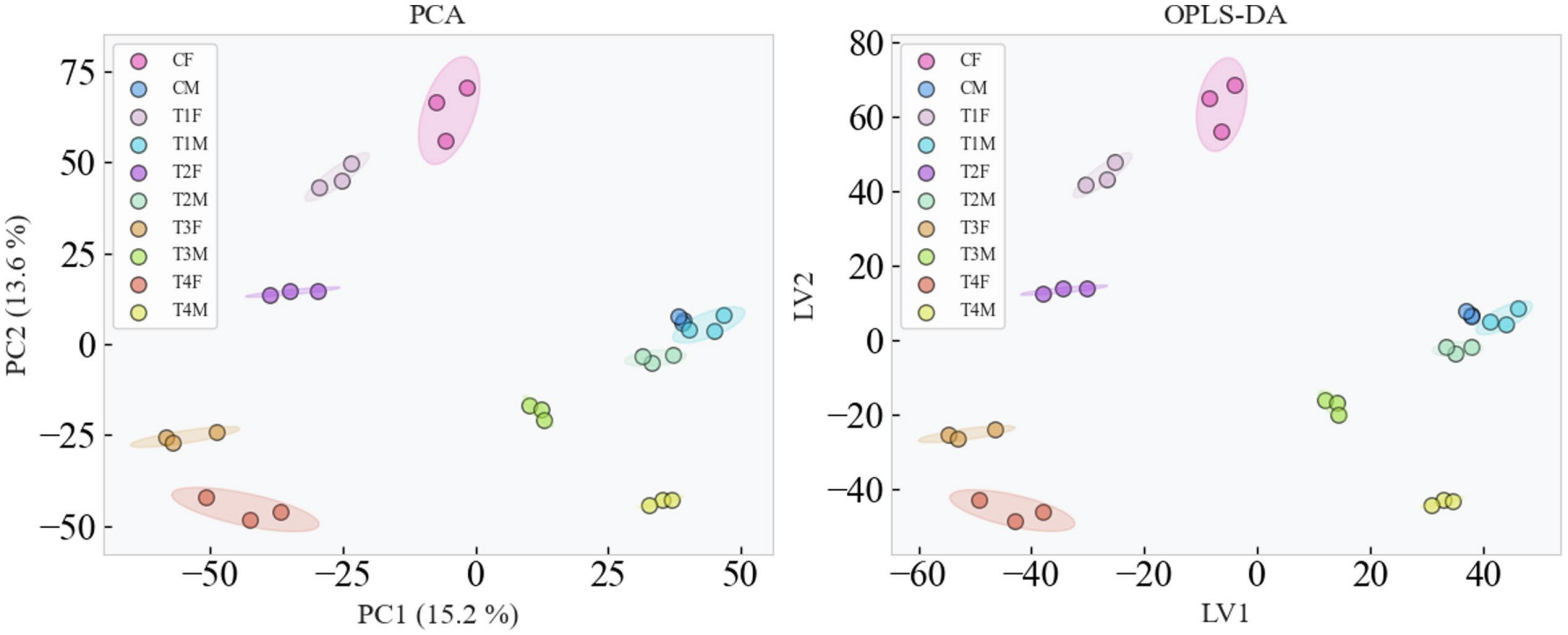
Metabolomic analysis: global metabolic profile assessed by principal component analysis (PCA) and orthogonal partial least squares-discriminant analysis (OPLS-DA).

To further unravel the downstream metabolic mechanisms through which the gut microbiota modulates gender-specific physiological outcomes of L-SeMC, we performed untargeted metabolomics profiling of intestinal contents. The results are summarized in Figure 11, with detailed in Supplementary Tables S2–S5 showing differential metabolites categorized by bile acids, free fatty acids (FFAs), tryptophan derivatives, and other metabolites. This analysis establishes a critical link between microbial dysbiosis, host metabolic perturbation, and hepatic injury, thereby reinforcing the multi-omics mechanistic pathway proposed in this study.

Figure 11 demonstrates clear gender-specific and dose-dependent metabolic separation in the metabolic profiles. Both PCA and PLS-DA analyses showed that samples from high dose (T4) female rats formed a distinct cluster, separated from control and low-to-moderate dose (T1-T3) groups along the PC1 (15.2%) and LV1 axes. This indicates profound metabolic dysregulation in T4 females. In contrast, samples from T2 males showed partially overlap with those from those from T1 male group. This metabolic stability aligns with their stable gut microbiota and intact hepatic phenotype, confirming host metabolism as a key intermediate in the gut microbiota-hepatic axis.

**Figure 12:**
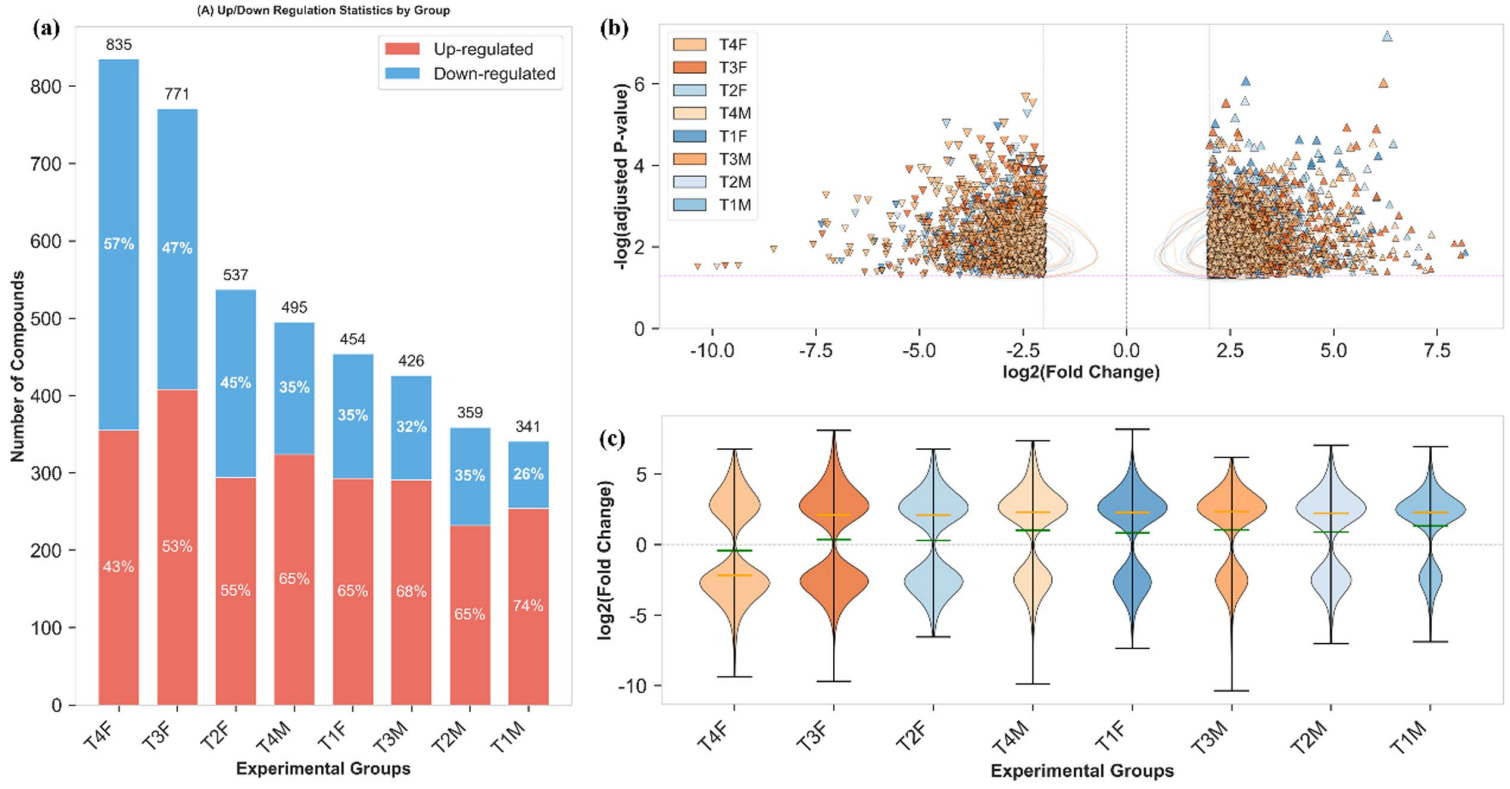
Differential gut metabolomic profiling in rats following oral L-SeMC administration. **(a)** Statistical summary of upregulated and downregulated metabolites; **(b)** Distribution of differential gut metabolites plotted as logFC vs. significance, with 2D kernel density estimates (KDE). Semi-transparent contour lines (for groups with >10 significant compounds) connect points of equal probability density: inner contours indicate regions of highest compound density, outer contours denote sparser distributions, elongated shapes reflect correlated logFC and significance, and circular shapes indicate uniform distribution. Contours complement scatter points to visualize overall trends without obscuring individual data. Upright triangles denote upregulated metabolites, while inverted triangles represent downregulated metabolites. **(c)** Distribution of differential gut metabolites across experimental groups, the blue line represents the mean of log_2_(FC) values, and the purple line represents the median.

Gut metabolomic profiling (Figure 12) also displayed a gender-specific and dose-dependent manner that closely aligns with the metabolic separation observed in Figure 11. Specially, female rats displayed greater disturbance males within the same dose group, and higher dose of L-SeMC induced more pronounced metabolic alteration compared to lower doses. Among all groups, T4 females had one of the highest numbers of differential metabolites, with a nearly balanced 43% upregulation and 57% downregulation (Figure 12a) as well as marked significant perturbations (Figure 12b). In contrast, metabolically stable groups (e.g., T1/T2 males) had far fewer differential metabolites (341 in T1 males) and a strong 74% upregulation bias, reflecting targeted shifts that support homeostasis. Consistently, heterogeneity in metabolic shifts mirrored these trends: T4 females showed a broad, dispersed logFC distribution, while T1 males exhibited a narrow, concentrated profile (Figure 12c).

Detailed metabolite analysis (Supplementary Tables S2–S5) reveals functionally critical dose-specific and gender-dependent changes. For bile acids (Supplementary Table S2), Low-to-moderate doses (T1-T3) induced beneficial alterations. T3 female rats were enriched with hepatoprotective and anti-inflammatory bile acids (e.g., ursodeoxycholic acid, taurolithocholic acid), whereas T3 male rats maintained elevated levels of glycochenodeoxycholic acid (logFC = 4.297), which is involved in lipid homeostasis. In contrast, T4 female rats exhibited significant downregulation of protective bile acids (e.g., deoxycholic acid, chenodeoxycholic acid, and beta-muricholic acid) alongside an abnormal accumulation of sulfated conjugates, indicating impaired enterohepatic circulation and hepatic lipid metabolism. For FFAs (Supplementary Table S3), T3 rats enriched gut health-promoting (e.g., stearidonic acid in females and stearic acid in males) and anti-inflammatory FFAs, whereas T4 female rats were depleted of anti-inflammatory and barrier-enhancing FFAs (e.g., dihomo-alpha-linolenic acid and docosa-4,10,13,16-tetraenoic acid). Tryptophan derivatives (Supplementary Table S4) further highlighted this dose dichotomy. T3 females accumulated beneficial indole metabolites like indole 3-ethanol. In stark contrast, T4 females showed an abnormally overaccumulation of indole metabolites (e.g., indole-3-carboxylic acid, logFC = 5.93; indole 3-ethanol, logFC = 5.94) and a depletion of protective peptides such as Asn-Trp-His. For other unclassified metabolites (Supplementary Table S5), T4 female rats showed downregulated glutathione (logFC = −2.906), a key hepatic detoxifier. In contrast, the T1-T3 doses enriched hepatoprotective metabolites like *γ*-tocotrienol and membrane-stabilizing phosphatidylserine (PS (18,0/20,4)).

Notably, these metabolic alterations are directly linked to the shifts in gut microbiota composition (Table 1). The enrichment of beneficial *Bifidobacterium* species (e.g., *Bifidobacterium cricetid* and *Bifidobacterium choerinum*) in T3 female rats correlates with the elevated levels of gut barrier-protective FFAs and hepatoprotective bile acids, consistent with the known role of *Bifidobacterium* in regulating fatty acid metabolism and bile acid conjugation. Conversely, the depletion of key functional taxa in T4 female rats, such as *Candidatus Arthromitus* sp. SFB mouse NL, *Phascolarctobacterium* sp. Marseille Q4147, and *Ruminococcus* sp. AF37 3 AC, directly contributes to the observed downregulation of protective bile acids and anti-inflammatory FFAs. These taxa are known to be involved in bile acid transformation and anti-inflammatory metabolite synthesis. In male rats, the modest microbial alterations (e.g., depletion of *Prevotella* species in T4 male rats) are paralleled by milder metabolic perturbations. Although T4 male rats show downregulation of deoxycholic acid (Supplementary Table S2) and glutathione (Supplementary Table S5), they retained enrichment of beneficial FFAs (e.g., alpha-linolenic acid) and tryptophan-derived peptides (e.g., Tyr-Trp-Ser), which confer a buffering effect against hepatic injury.

These integrated microbial-metabolic interactions provide a direct mechanistic explanation for the divergent physiological outcomes. In T4 female rats, the synergistic effects of bile acid dysregulation, depletion of anti-inflammatory FFAs, and abnormal tryptophan metabolism collectively amplify hepatic inflammation and parenchymal injury, aligning with the elevated ALT/AST and corresponding histological damage. In contrast, male rats maintain metabolic homeostasis through the retention of beneficial metabolites, which underscores their relative resistance to high-dose L-SeMC-induced hepatotoxicity. Collectively, the findings demonstrate that L-SeMC modulates gut microbiota-metabolite-liver axis in a gender-specific manner. Low-to-moderate doses (T1-T3) promote a protective “microbe-beneficial metabolite” axis that supports hepatic health, whereas high-dose exposure disrupts this axis only in females, leading to metabolic dysregulation and subsequent hepatic injury. This confirms the gut microbiota-metabolite-liver axis as a core mediator of the gender-biased effects of L-SeMC.

### 3.6 Correlation of differential gut microbiota and metabolites with food intake and body weight

**Figure 13:**
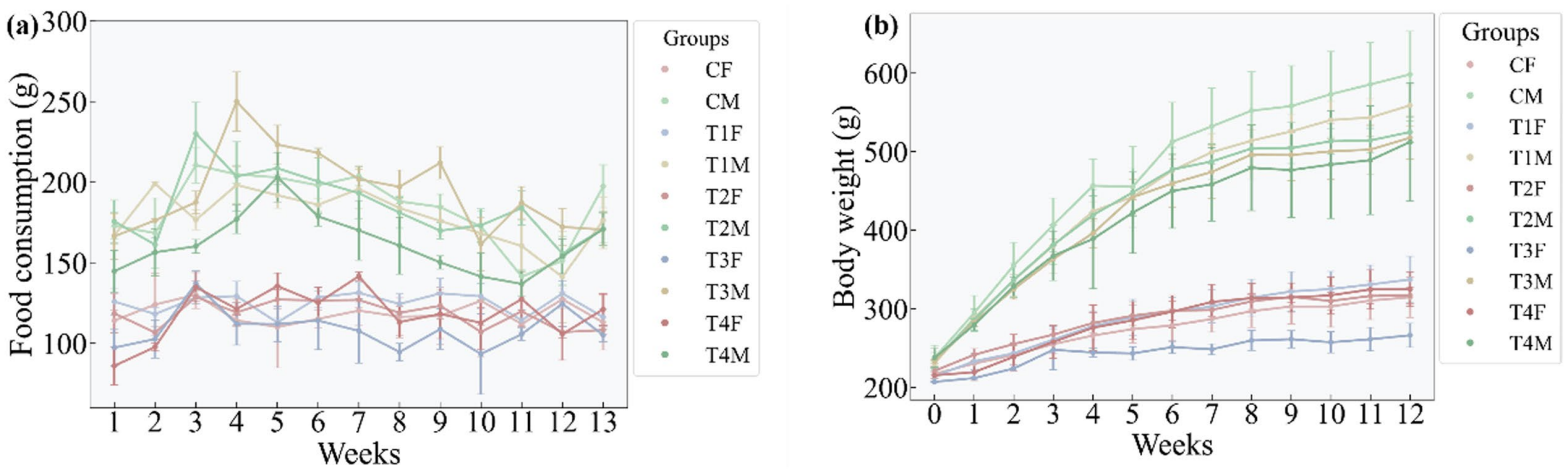
The food intakes and the body weights of rats in the experiment period. **(a,b)** represent the food intakes of the rat and the body weight of rats, respectively, in the experiment duration.

Throughout the entire experimental period, the food intake and body weight of male rats were significantly higher than those of female rats (Figures 13a,b), an observation consistent with the well-established notion that male rats typically exhibit substantially greater food intake and higher body weights than females. Notably, no significant differences in food intake were detected among male rats across the study. Regarding body weight, female rats in the T3 group exhibited the lowest body weight, while those in the females in the T1 group had the highest; in male rats, the T4 group showed the lowest body weight, whereas the CM group exhibited the highest.

**Figure 14:**
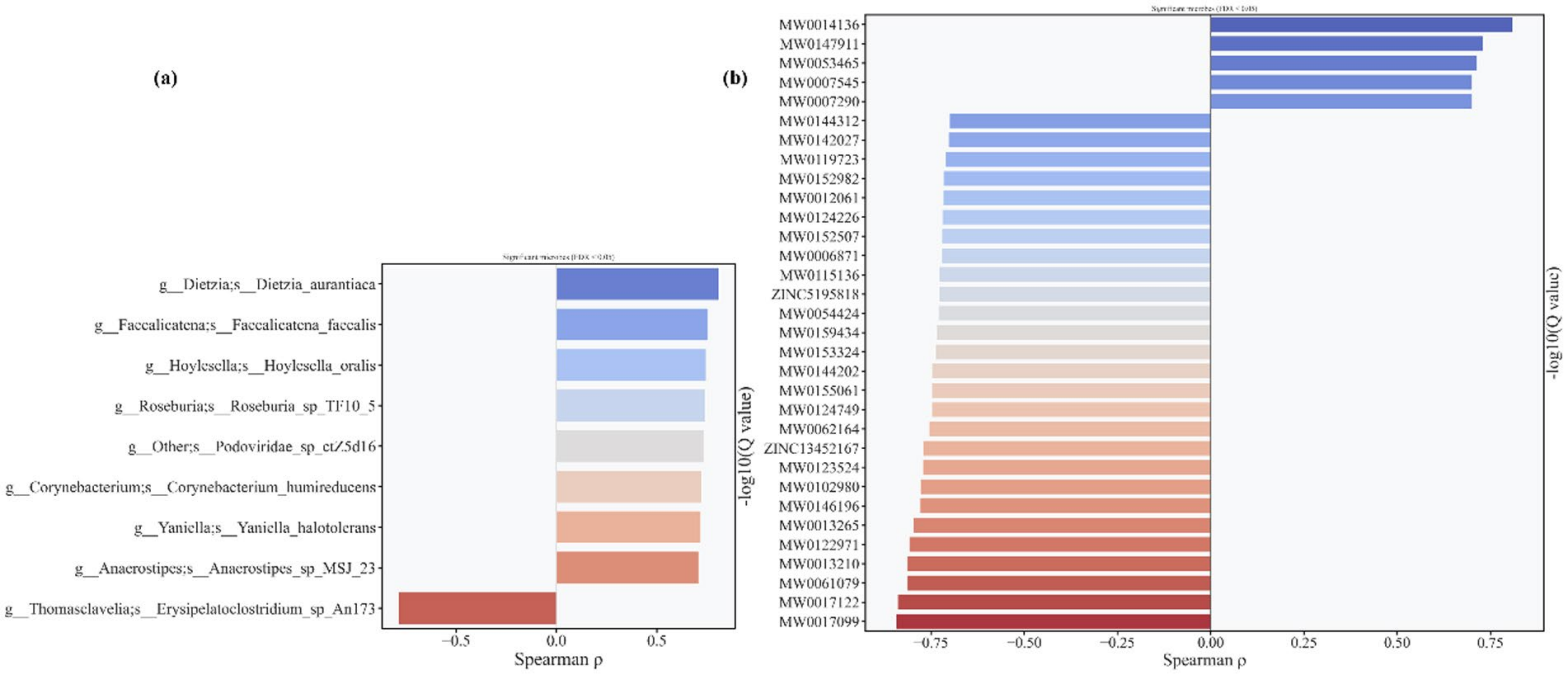
The correlation between differential microorganisms **(a)** and differential metabolites **(b)** with body weights of rats. Only those differential microorganisms and metabolites exhibiting a Spearman correlation coefficient over 0.7 are presented.

To clarify whether the identified differential gut microbiota and metabolites are link to L-SeMC-induced physiological phenotypes through the regulation of energy balance, we analyzed their Spearman correlations with weekly food intake and body weight (Figure 14). Notably, no significant correlations were observed between food intake and either differential microbial taxa or metabolites across all groups. This indicates that L-SeMC-induced alterations in the gut ecosystem are independent of changes in food consumption, ruling out food consumption as a confounding factor in mediating microbial and metabolic perturbations.

Notably, 9 differential microbial taxa (Figure 14a) and 32 differential metabolites (Figure 14b) were highly correlated with rat body weight. This result highlights that while microbial and metabolic alterations induced by L-SeMC are not associated with food intake, a specific subset of these gut-derived factors is tightly associated with body weight, suggesting their potential role in weight regulation under L-SeMC exposure. Beyond the established “gut microbiota-metabolite-liver” axis, this association uncovers an additional regulatory layer, thereby enhancing our multi-level mechanistic understanding of L-SeMC’s physiological effects. Furthermore, these body weight-associated microbes and metabolites may further contribute to the observed gender-specific responses, consistent with the gender-specific microbial and metabolic perturbations identified earlier.

### 3.7 The potential injury of oral L-SeMC administration on the metal status of rats

**Figure 15:**
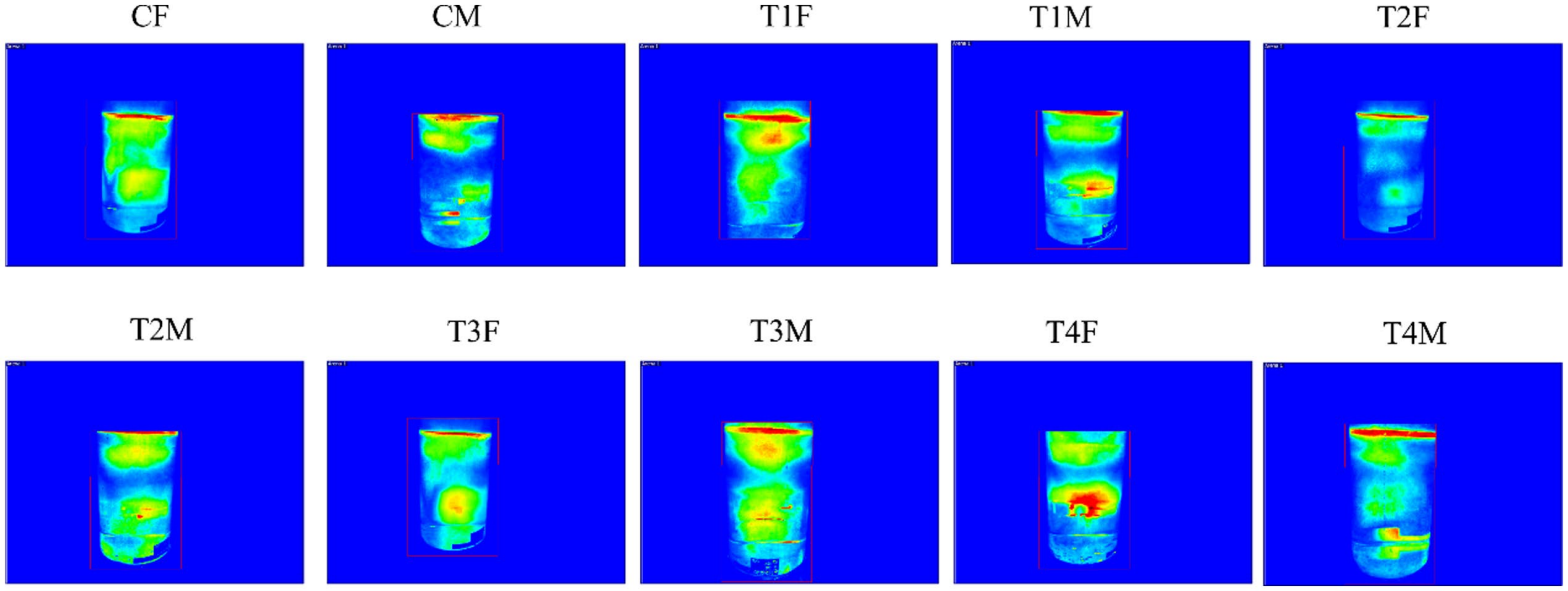
Representative thermal imaging maps of rats from all experimental groups during the forced swimming test. CF, T1T, T2F, T3F, and T4F represent the female subgroups of the control, T1, T2, T3, and T4 groups, respectively. CM, T1M, T2M, T3M, and T4M denote the male subgroups of the control, T1, T2, T3, and T4 groups, respectively.

Given the role of L-SeMC in modulating anti-inflammatory metabolites in the gut, we further investigated whether its administration exerts adverse effects on the mental state of rats using the forced swimming test. The results were shown in Figure 15. Analysis of locomotor activity patterns, characterized by thermal signal distribution and regional intensity, revealed that all L-SeMC-treated groups (T1-T4) exhibited patterns consistent with the control groups. No overt reductions or aberrant patterns of activity, which would indicate adverse mental state alterations, were observed at any dose. Therefore, under the conditions of this study, L-SeMC exposure did not induce significant changes in the behavioral measures of mental state in rats. This finding is consistent with modulatory, rather than disruptive, role of L-SeMC on gut-derived anti-inflammatory metabolites, indicating that these microbial metabolic changes did not translate into detrimental neurobehavioral outcomes.

## 4 Discussion

Selenium, an essential trace element for mammals, exhibits a safety profile highly dependent on its chemical form, dosage, exposure duration, and the specific biological model (8, 21). For dietary supplements, selenium-enriched yeast has been confirmed safe at doses below 1.5 mg Se/kg (30), whereas selenium nanoparticles show no significant cytotoxicity in healthy L929 cells at concentrations below 5 mg/mL (31). Furthermore, the glucosamine-selenium conjugate exhibits an LD_50_ of 92.31 mg/kg bw/day in ICR mice; a 30-day subchronic toxicity study further confirmed that doses below 9.23 mg/kg bw/day do not induce hepatic or renal toxicity (32); Additionally, 12 μmol/L of selenium-enriched peptides exert a robust protective effect on human L-02 hepatocytes against ethanol-induced injury (33). Yang et al. (34) proposed an acceptable daily intake (ADI) of 3.4 μg/kg bw/day for Se-methylselenocysteine. Park et al. (29) reported that sodium dichloroisocyanurate at 2.0 mg/m^3^ is non-toxic to rats of both sexes.

Regarding L-SeMC specifically, Yu et al. (22) reported a NOAEL (no observed adverse effect level) of 0.15 mg/kg bw/day for male rats and 0.30 mg/kg bw/day for female rats. Notably, these NOAEL values are substantially lower than those reported for selenium-enriched peptides (0.6 mg/kg bw/day), selenium yeast (0.30 mg/kg bw/day), and even sodium selenite (0.30 mg/kg bw/day). Furthermore, the finding that female rats have a higher safe dose threshold than male rats conflicts with prior literature (21, 35) and with the female-biased susceptibility observed in our study. Additionally, the reported higher safe dose for sodium selenite compared to the organic form L-SeMC appears to contradict the well-established consensus that organic selenium compounds generally possess superior safety profiles. These discrepancies highlight the complexity of gender-specific toxicity for selenium-containing compounds. A potential contributing factor is the baseline selenium level in the experimental diet. Our analysis revealed a background selenium concentration of 104 mg/kg in the rat diet (see Supplementary 1, Supplementary Figure S6), which may have influenced the effective dose and toxicity outcomes in the cited study.

The integrated multi-omics data highlight the gut microbiota-metabolite axis as a central mechanism mediating the dose- and gender-dependent biological effects of L-SeMC, linking its exposure to either beneficial or toxic outcomes. At low-to-moderate doses of L-SeMC (0.25–0.75 mg/kg bw/day in rats, equivalent to approximately 1.04–3.12 mg/60 kg bw/day for human), L-SeMC promotes intestinal health through beneficial remodeling of the gut ecosystem. Microbial profiling showed these doses enrich beneficial taxa such as *Bifidobacterium* species, which are known to enhance intestinal barrier integrity and immune modulation (36, 37). Concurrently, metabolome analysis revealed a favorable metabolic shift in the metabolic landscape, characterized by the enrichment of hepatoprotective bile acids (e.g., ursodeoxycholic acid, glycochenodeoxycholic acid), an increase in anti-inflammatory FFAs (e.g., stearidonic acid), and the accumulation of beneficial indole derivatives (e.g., indole 3-ethanol) (Supplementary Tables S2–S4). These metabolites act synergistically to reinforce the intestinal and systemic homeostasis: bile acids regulate gut epithelial proliferation and microbial composition (38); anti-inflammatory fatty acids suppress pro-inflammatory cytokine production (e.g., TNF-*α*, IL-6) and promote anti-inflammatory signaling (39); and indole derivatives modulate mucosal immunity and help prevent intestinal inflammation (40). Collectively, this “microbe-beneficial metabolites” axis activated by low-to-moderate L-SeMC not only directly improves intestinal health but also maintains systemic metabolic balance via the gut-liver axis, explaining the systemic protective effects of L-SeMC.

Notably, the T3 dose (1.5 mg/kg bw/day in rats, equivalent to 6.24 mg/60 kg bw/day in humans) represents a critical threshold where the gut microbiota-metabolite axis transitions from a beneficial to a perturbed state. Whereas T3 may retain partial beneficial effects (e.g., enrichment of anti-inflammatory metabolites), it simultaneously induces a significant reduction in gut microbial alpha diversity. This loss of diversity signals an initial decline in microbial community resilience, which is often associated with reduced functional redundancy—the ecosystem’s capacity to maintain metabolic stability in the face of environmental stress (41). In the context of our study, this compromised resilience weakens the gut microbiota’s ability to buffer against the excessive L-SeMC exposure, resulting in subtle dysregulation of key metabolic pathways, including shifts in bile acid conjugation and tryptophan metabolism. Although these alterations did not culminate in overt toxicity within 90-day study, they indicate a precarious metabolic state that may predispose the host to adverse outcomes upon chronic or higher-dose exposure. This finding highlights the importance of incorporating gut microbial diversity metrics into safety assessments, as it provides an early warning signal of ecological disruption that precedes detectable injury via conventional toxicological endpoints.

High-dose L-SeMC (2.25 mg/kg bw/day in rats, equivalent to 9.36 mg/60 kg bw/day in humans) severely disrupts the gut microbiota-metabolite axis, with particularly pronounced effects in female rats, culminating in systemic toxicity. Our microbiome data demonstrate that this exposure depletes protective taxa such as *Candidatus Arthromitus*, *Phascolarctobacterium*, and *Ruminococcus* species, which are involved in bile acid transformation, short-chain fatty acid production, and anti-inflammatory metabolite synthesis (42). This microbial depletion directly translates to profound metabolic dysregulation. In female rats, this manifests a marked downregulation of protective bile acids (e.g., deoxycholic acid, chenodeoxycholic acid, and *β*-muricholic acid) and anti-inflammatory FFAs (e.g., dihomo-*α*-linolenic acid), coupled with an abnormal overaccumulation of indole pathway intermediates and a depletion of protective tryptophan-derived peptides. Collectively, these disturbances compromise intestinal barrier integrity, activate hepatic inflammatory signaling pathways, and induce oxidative stress (evidenced by downregulation of glutathione, a key antioxidant metabolite). These cascading events ultimately synergize to drive the irreversible hepatosplenic damage observed in high-dose females. Notably, this female-specific injury is closely associated with estrogen, a key sex hormone regulating the gut microbiota-metabolite axis. Physiologically, estrogen in females promotes beneficial gut taxa (e.g., *Bifidobacterium*) and protective metabolites, while high-dose L-SeMC disrupts hepatic estrogen metabolism, leading to excessive unmetabolized estrogen that inhibits protective taxa and induces oxidative stress, synergizing with microbial dysregulation to accelerate hepatosplenic injury. Consistent with our histopathological scoring results, the obvious hepatic interstitial expansion and splenic enlargement in T4 group female rats were positively correlated with the corresponding metabolic dysregulation and increased spleen weight, further confirming the female-specific hepatosplenic toxicity induced by high-dose L-SeMC.

Importantly, the inherent resistance of male rats to high-dose L-SeMC-induced toxicity is similarly governed by the gut microbiota–metabolite axis. In contrast to females, males maintain a relatively stable gut microbial composition even at high doses, with only modest depletion of non-essential taxa like *Prevotella* species. This microbial stability underpins the preservation of a beneficial metabolite profile: male rats retain enrichment of anti-inflammatory FFAs and protective tryptophan-derived peptides (e.g., Tyr-Trp-Ser), which collectively help maintain intestinal barrier integrity and mitigate hepatic inflammation responses. Additionally, male-specific differences in bile acid metabolism may further enhance their resilience. Notably, male rats have lower endogenous estrogen levels, and their gut microbiota is less sensitive to estrogen-mediated regulation, which partially explains their higher resilience to high-dose L-SeMC-induced toxicity. Collectively, our findings indicate that estrogen is a key mediator linking female sex to L-SeMC-induced toxicity, and the disruption of estrogen metabolism and its regulatory effect on the gut microbiota-metabolite axis are important mechanisms underlying female-specific injury. These gender-specific differences in the gut microbiota-metabolite axis fundamentally explain the severe toxicity observed in high-dose females and its absence in males, thereby establishing this axis as a central determinant of the gender-biased physiological responses to selenium compounds.

The central role of the gut microbiota-metabolite axis in mediating L-SeMC’s effects also provides a framework to reconcile the discrepancies with the findings reported by Yu et al. (22). Their study relied on traditional toxicity endpoints such as serum biochemistry and gross pathology, without evaluating gut microbial or metabolic perturbations. This methodological gap likely led them to miss the subtle, dose- and gender-specific shifts within this critical axis. For instance, their reported higher NOAEL in females may correspond to the protective, low-dose phase of L-SeMC’s action on the gut ecosystem (consistent with our observations in T1-T2 groups), while their protocol would not have captured the axis’s eventual collapse in females at higher doses. Furthermore, differences in experimental conditions, such as the background selenium level in the diet, could fundamentally alter the baseline state of the gut microbiota. A high dietary selenium concentration might precondition the microbial community, potentially increasing its sensitivity to L-SeMC and thereby leading to the lower apparent NOAELs observed in males in their study.

In summary, our findings establish the gut microbiota-metabolite axis as the central functional link between L-SeMC exposure and its biological effects. The effects are strictly dose- and gender-dependent: low-to-moderate doses (0.25–0.75 mg/kg bw/day in rats; equivalent to1.04–3.12 mg/60 kg bw/day in humans) reinforce this axis by enriching beneficial microbes and their derived metabolites, thereby promoting intestinal health and systemic homeostasis. The T3 dose (1.5 mg/kg bw/day in rats; equivalent to 6.24 mg/60 kg bw/day in humans) represents a critical transition point, where microbial diversity is compromised, signaling a need for cautious application. In contrast, high-dose exposure (2.25 mg/kg bw/day in rats; equivalent to 9.36 mg/60 kg bw/day in humans) causes a collapse of this axis selectively in females, leading to profound metabolic dysregulation and irreversible hepatosplenic toxicity, a fate largely averted in males due to their more resilient gut ecosystem. This mechanistic framework not only explains the dose- and gender-specific outcomes of L-SeMC but also highlights the imperative to integrate gut microbiota and metabolomic profiling into the safety assessment of selenium-containing compounds. Such an integrated approach is essential for accurately delineating their beneficial-to-toxic dose window and for understanding their distinct risk profiles across biological sexes.

Finally, the following key conclusions were drawn:

1. Dose-dependent effects on gut ecology and safety window: Low-to-moderate doses of L-SeMC (0.25–0.75 mg/kg bw/day in rats; equivalent to 1.04–3.12 mg/60 kg bw/day in humans) promote intestinal health in rats by enriching beneficial gut microbial taxa. The T1-T2 dosage range is therefore identified as the safest for application. In contrast, the T3 dose (1.5 mg/kg bw/day in rats; equivalent to 6.24 mg/60 kg bw/day in humans) reduces gut microbial alpha diversity, indicating a transition point that necessitates cautious use with monitoring for gut ecological disruption.
2. Systemic protective mechanism via a core axis: At low-to-moderate doses, L-SeMC confers systemic protective benefits by activating a “gut microbiota-metabolite-liver” axis. The induced beneficial microbial shifts drive favorable metabolic adaptations, including the enrichment of hepatoprotective bile acids, anti-inflammatory fatty acids, and beneficial indole derivatives, which work synergistically to sustain hepatic function and overall physiological homeostasis.
3. Gender-specific high-dose toxicity mediated by axis collapse: High-dose L-SeMC (2.25 mg/kg bw/day in rats; equivalent to 9.36 mg/60 kg bw/day in humans) induces irreversible hepatosplenic injury exclusively in female rats. This gender-biased toxicity is mechanistically driven by the collapse of the gut microbiota-metabolite axis in females, leading to severe metabolic dysregulation. The relative stability of this axis in males underlies their resistance to high-dose toxicity.
4. Suggestions for preventing or minimizing gender-related toxicity: For future researches, gender-specific dose stratification should be adopted, with lower doses recommended for females to avoid gut microbiota-metabolite axis collapse and subsequent toxicity. Additionally, gut microbiota and metabolomic profiling should be integrated into routine safety monitoring to early identify subtle perturbations of the gut ecosystem, and probiotic intervention targeting beneficial taxa (e.g., *Bifidobacterium*) could be considered to enhance the resilience of the gut microbiota-metabolite axis in females, thereby reducing their susceptibility to selenium-related toxicity.

## Data Availability

The microbiome data of all groups are deposited in the National Center for Biotechnology Information (NCBI) database (https://www.ncbi.nlm.nih.gov/) access number: PRJNA1417577. Metabolome data for all groups are deposited in the National Genomics Data Center (NGDC) (https://ngdc.cncb.ac.cn/), access numbers: OMIX014882 and OMIX014883.

## References

[1] Schwarz K Foltz CM. Selenium as an integral part of factor 3 against dietary necrotic liver degeneration. J Am Chem Soc. (1957) 79:3292–3. doi: 10.1021/ja01569a087 10408880

[2] Rotruck JT Pope AL Ganther HE Swanson AB Hafeman D Hoekstra WG. Selenium: biochemical role as a component of glutathione peroxidase. Science. (1973) 179:588–90. doi: 10.1126/science.179.4073.588 4686466

[3] Jenkins DJA Kitts D Giovannucci EL Sahye-Pudaruth S Paquette M Mejia SB . Selenium, antioxidants, cardiovascular disease, and all-cause mortality: a systematic review and meta-analysis of randomized controlled trials. Am J Clin Nutr. (2020) 112:1642–52. doi: 10.1093/ajcn/nqaa245 33053149 PMC7727482

[4] Schomburg L Orho-Melander M Struck J Bergmann A Melander O. Selenoprotein-P deficiency predicts cardiovascular disease and death. Nutrients. (2019) 11:1852. doi: 10.3390/nu11081852, 31404994 PMC6723215

[5] Shu YY Zhu YJ Li WG. Protective role of selenium against hepatitis B virus and primary liver cancer in Qidong. Biol Trace Elem Res. (1997) 56:117–24. doi: 10.1007/BF02778987, 9152515

[6] Hatfield DL Tsuji PA Carlson BA Gladyshev VN. Selenium and selenocysteine: roles in cancer, health, and development. Trends Biochem Sci. (2014) 39:112–20. doi: 10.1016/j.tibs.2013.12.007, 24485058 PMC3943681

[7] Khosravi M Poursaleh A Ghasempour G Shaikhnia F Najafi M. The effects of oxidative stress on the development of atherosclerosis. Biol Chem. (2019) 400:711–32. doi: 10.1515/hsz-2018-0397, 30864421

[8] Rayman MP. Selenium and human health. Lancet. (2012) 379:1256–68. doi: 10.1016/s0140-6736(11)61452-9, 22381456

[9] Koyama H Omura K Ejima A Kasanuma Y Watanabe C Satoh H. Separation of selenium-containing proteins in human and mouse plasma using tandem high-performance liquid chromatography columns coupled with inductively coupled plasma-mass spectrometry. Anal Biochem. (1999) 267:84–91. doi: 10.1006/abio.1998.2949, 9918658

[10] Bai S Zhang M Tang S Li M Wu R Wan S . Effects and impact of selenium on human health, a review. Molecules. (2024) 30:50. doi: 10.3390/molecules30010050, 39795109 PMC11721941

[11] Hall JA Van Saun RJ Bobe G Stewart WC Vorachek WR Mosher WD . Organic and inorganic selenium: I. Oral bioavailability in ewes1. J Anim Sci. (2012) 90:568–76. doi: 10.2527/jas.2011-4075 21965451

[12] Pehrson B Knutsson M Gyllenswärd M. Glutathione peroxidase activity in heifers fed diets supplemented with organic and inorganic selenium compounds. J Food Compos Anal. (1989) 19:53–6. doi: 10.1016/j.jfca.2018.05.005

[13] Tung YC Tsai ML Kuo FL Lai CS Badmaev V Ho CT . Se-methyl-L-selenocysteine induces apoptosis via endoplasmic reticulum stress and the death receptor pathway in human colon adenocarcinoma COLO 205 cells. J Agric Food Chem. (2015) 63:5008–16. doi: 10.1021/acs.jafc.5b01779, 25943382

[14] Babaer D Zheng M Ivy MT Zent R Tiriveedhi V. Methylselenol producing selenocompounds enhance the efficiency of mammaglobin-a peptide vaccination against breast cancer cells. Oncol Lett. (2019) 18:6891–8. doi: 10.3892/ol.2019.11010, 31807192 PMC6876340

[15] Zeng H Cheng WH Johnson LK. Methylselenol, a selenium metabolite, modulates p53 pathway and inhibits the growth of colon cancer xenografts in Balb/c mice. J Nutr Biochem. (2013) 24:776–80. doi: 10.1016/j.jnutbio.2012.04.008, 22841391

[16] Medina D Thompson H Ganther H Ip C. Se-methylselenocysteine: a new compound for chemoprevention of breast cancer. Nutr Cancer. (2001) 40:12–7. doi: 10.1207/s15327914nc401_5, 11799917

[17] Yang X Wang M Kang X Mo F Si P Ma J . L-se-methylselenocysteine loaded mucoadhesive thermogel for effective treatment of vulvar candidiasis. Int J Pharm. (2022) 622:121851. doi: 10.1016/j.ijpharm.2022.121851, 35618178

[18] Maov W Liu Y Gu W Xu W Liu J Ling Q . Se-Methylselenocysteine ameliorates DEHP-induced ferroptosis in testicular sertoli cells via the Nrf2/GPX4 axis. Environ Toxicol. (2025) 40:191–203. doi: 10.1002/tox.24425 39360521

[19] Xie Y Liu Q Zheng L Wang BT Qu X Ni J . Se-methylselenocysteine ameliorates neuropathology and cognitive deficits by attenuating oxidative stress and metal dyshomeostasis in alzheimer model mice. Mol Nutr Food Res. (2018) 62:e1800107. doi: 10.1002/mnfr.201800107, 29688618

[20] Ban B Yang H Liu Y Luo Z. Se-methylselenocysteine inhibits migration and glycolysis in anaplastic thyroid carcinoma cells via the ERK1/2 signaling pathway in vitro. Ann Clin Lab Sci. (2024) 54:810–9. 39855728

[21] Fairweather-Tait SJ Bao Y Broadley MR Collings R Ford D Hesketh JE . Selenium in human health and disease. Antioxid Redox Signal. (2011) 14:1337–83. doi: 10.1089/ars.2010.3275 20812787

[22] Yu L Li Y Qu W Zheng Y Chen X Fu S . Systemic subchronic toxicity and comparison of four selenium nutritional supplements by 90-day oral exposure in Sprague-dawley rats. Food Chem Toxicol. (2023) 181:114059. doi: 10.1016/j.fct.2023.114059, 37758048

[23] Verma AK Kumar A Rahal A Kumar V Roy D. Inorganic versus organic selenium supplementation: a review. Pak J Biol Sci. (2012) 15:418–25. doi: 10.3923/pjbs.2012.418.425, 24163951

[24] Takahashi K Suzuki N Ogra Y. Effect of administration route and dose on metabolism of nine bioselenocompounds. J Trace Elem Med Biol. (2018) 49:113–8. doi: 10.1016/j.jtemb.2018.05.007, 29895359

[25] Takahashi K Suzuki N Ogra Y. Effect of gut microflora on nutritional availability of selenium. Food Chem. (2020) 319:126537. doi: 10.1016/j.foodchem.2020.126537, 32193059

[26] Song J Lin H. Experimental study on promotion of peripheral nerve regeneration by selenium-methylselenocysteine. Zhonghua Xiu Fu Chong Jian Wai Ke Za Zhi. (2024) 38:598–607. doi: 10.7507/1002-1892.202402031 PMC11096885 38752248

[27] Stallings MT Cardon BR Hardman JM Bliss TA Brunson SE Hart CM . A high isoflavone diet decreases 5′ adenosine monophosphate-activated protein kinase activation and does not correct selenium-induced elevations in fasting blood glucose in mice. Nutr Res. (2014) 34:308–17. doi: 10.1016/j.nutres.2014.03.003 24774067

[28] Organization for Economic Cooperation and Development. Test No. 413: Subchronic Inhalation Toxicity: 90-day Study, OECD Guidelines for the Testing of Chemicals, Section 4. Paris: OECD Publishing (2018). Available online at: https://www.oecd.org/en/publications/test-no-413-subchronic-inhalation-toxicity-90-day-study_9789264070806-en.html

[29] Park CM Jeon S Kim YH Kim J Choi SJ Shim I . Sodium dichloroisocyanurate toxicity in rats during a 90-day inhalation toxicity study. Toxicol Appl Pharm. (2022) 456:116279. doi: 10.1016/j.taap.2022.116279, 36243099

[30] Li H Liu H Tang X Deng Z Li H. From soil to table: a comprehensive review of selenium-fortified foods. Compr Rev Food Sci Food Saf. (2025) 24:e70250. doi: 10.1111/1541-4337.70250, 40778620

[31] Cinar-Acar B. Size reduction of selenium nanoparticles synthesized from yeast beta glucan using cold atmospheric plasma. Sci Rep. (2025) 15:25875. doi: 10.1038/s41598-025-09192-8, 40670484 PMC12267570

[32] Che X Shang X Xing M Wei H Li W Li Z . Selenium-enriched *Lactiplantibacillus plantarum* alleviates alkalinity stress-induced selective hepatic insulin resistance in common carp. Int J Biol Macromol. (2025) 305:141204. doi: 10.1016/j.ijbiomac.2025.141204, 39986514

[33] Ling R Du C Li Y Wang S Cong X Huang D . Protective effect of selenium-enriched peptide from *Cardamine violifolia* on ethanol-induced L-02 hepatocyte injury. Biol Trace Elem Res. (2025) 203:139–52. 38538964 10.1007/s12011-024-04159-8

[34] Yang H Jia X. Safety evaluation of se-methylselenocysteine as nutritional selenium supplement: acute toxicity, genotoxicity and subchronic toxicity. Regul Toxicol Pharmacol. (2014) 70:720–7. doi: 10.1016/j.yrtph.2014.10.014, 25444999

[35] Rayman MP. The use of high-selenium yeast to raise selenium status: how does it measure up? Br J Nutr. (2004) 92:557–73. doi: 10.1079/BJN20041251 15522125

[36] Laursen MF. *Bifidobacterium infantis*-a key (late) colonizer of the infant gut? mSphere. (2026):e0085125. doi: 10.1128/msphere.00851-25 41586590 PMC12931266

[37] Ahmadi S Hasani A Yasdchi M Hasani A Poortahmasbe V Sedaghat FR . Altered gut microbiota, SCFAs, and barrier integrity markers in Alzheimer's and Parkinson's disease patients. Lett Appl Microbiol. (2026) 79:ovag010. doi: 10.1093/lambio/ovag010, 41591395

[38] Li T Chiang JY. Bile acid signaling in metabolic disease and drug therapy. Pharmacol Rev. (2014) 66:948–83. doi: 10.1124/pr.113.008201, 25073467 PMC4180336

[39] Srivastav H Kumar A Bhargawa PK Vishnoi N Kumar R. Probiotics: a promising microbial functional food with diverse health benefits (Online). Probiotics Antimicro. (2026). doi: 10.1007/s12602-025-10894-8 41586988

[40] Alexeev EE Lanis JM Kao DJ Campbell EL Kelly CJ Battista KD . Microbiota-derived indole metabolites promote human and murine intestinal homeostasis through regulation of interleukin-10 receptor. Am J Pathol. (2018) 188:1183–94. doi: 10.1016/j.ajpath.2018.01.011, 29454749 PMC5906738

[41] Louca S Polz MF Mazel F Albright MBN Huber JA O’Connor MI . Function and functional redundancy in microbial systems. Nat Ecol Evol. (2018) 2:936–43. doi: 10.1038/s41559-018-0519-1 29662222

[42] Lee JY Arai H Nakamura Y Fukiya S Wada M Yokota A. Contribution of the 7β-hydroxysteroid dehydrogenase from *Ruminococcus gnavus* N53 to ursodeoxycholic acid formation in the human colon. J Lipid Res. (2013) 54:3062–9306. doi: 10.1194/jlr.M039834, 23729502 PMC3793610

