# Supplementary material for: Gender-specific and dose-dependent responses to L-se-methylselenocysteine are mediated by the gut microbiota-metabolite axis: implications for intestinal homeostasis and safe clinical application
Source: Front Nutr. 2026 Mar 19;13:1803630. doi: 10.3389/fnut.2026.1803630 (PMC13045510; doi:10.3389/fnut.2026.1803630)
Supplement: Supplementary file 5 [file Data_Sheet_1.docx]

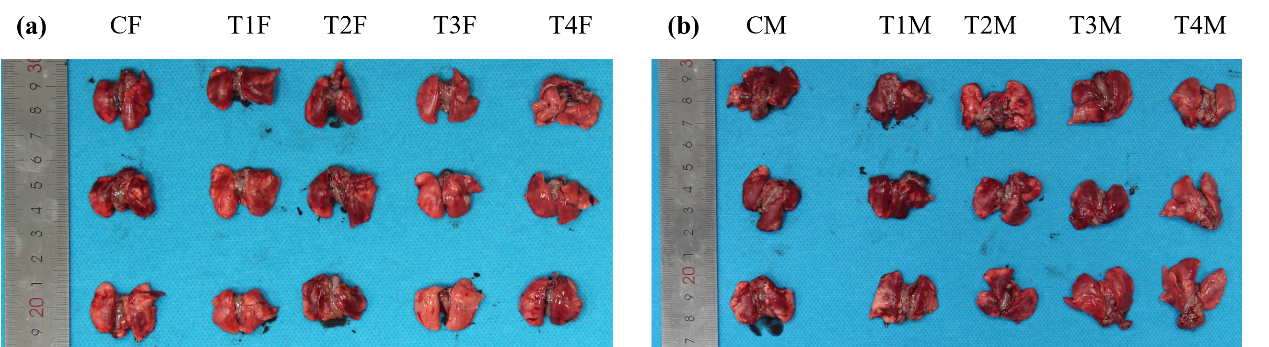


S1 The effects of oral L-SeMC on the lung morphology in female (a) and male (b) rats


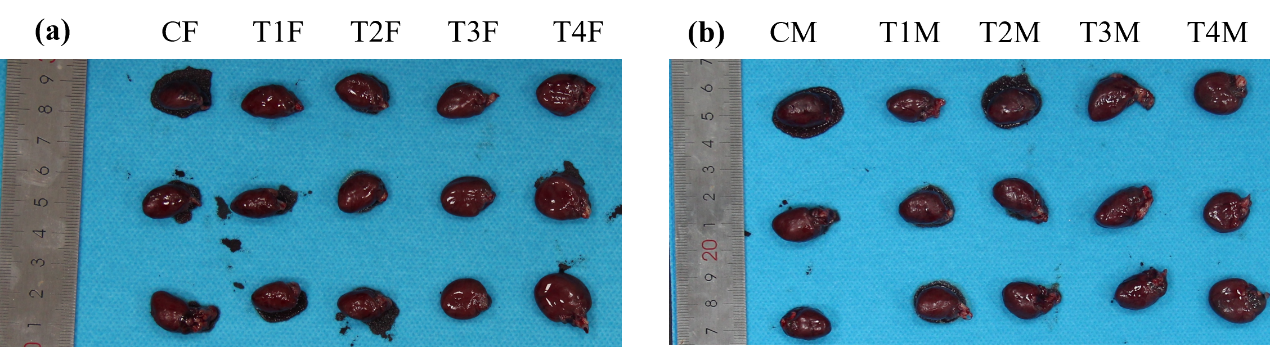


S2 The effects of oral L-SeMC on the heat morphology in female (a) and male (b) rats


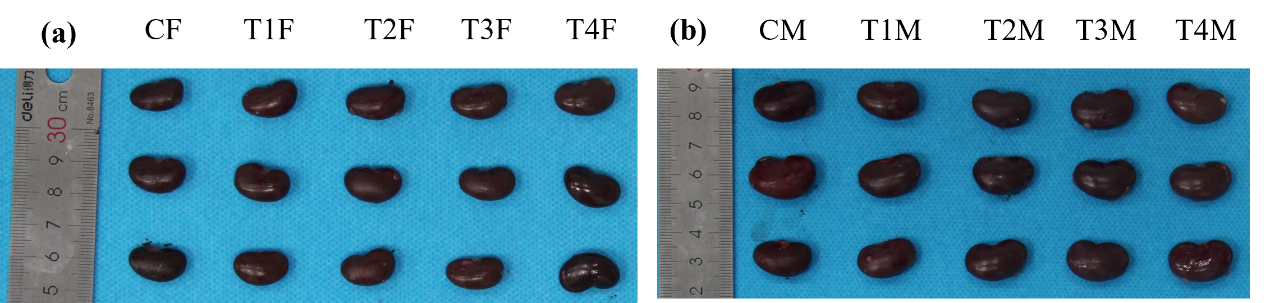


S2 The effects of oral L-SeMC on the kidney morphology in female (a) and male (b) rats


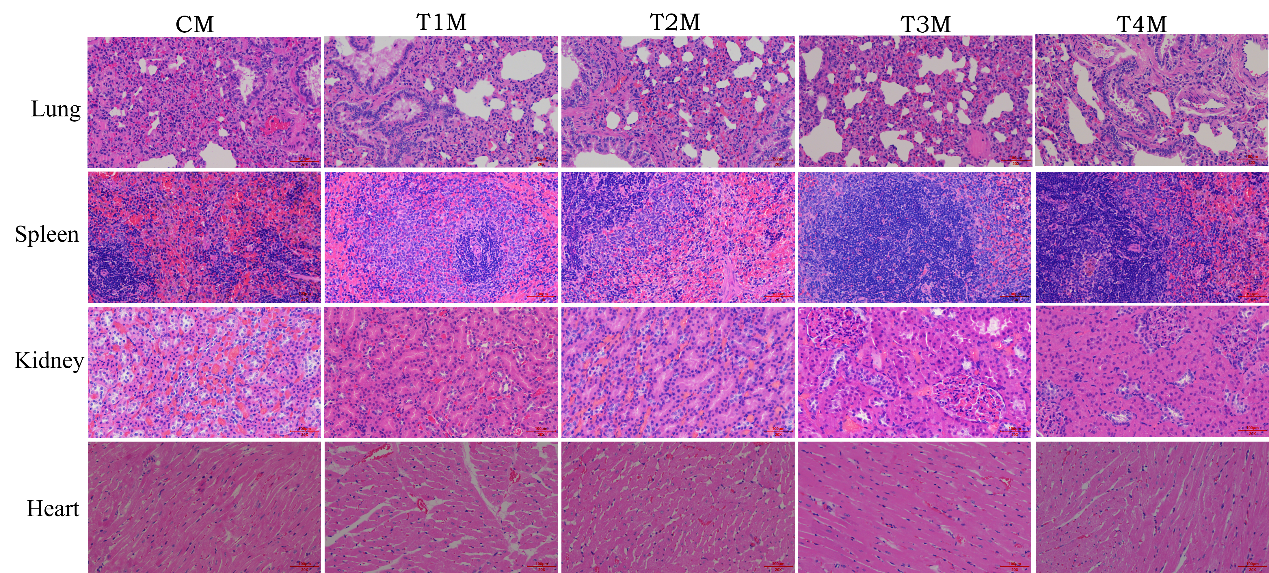


S4 The Hematoxylin-eosin staining of livers tissues from male rats


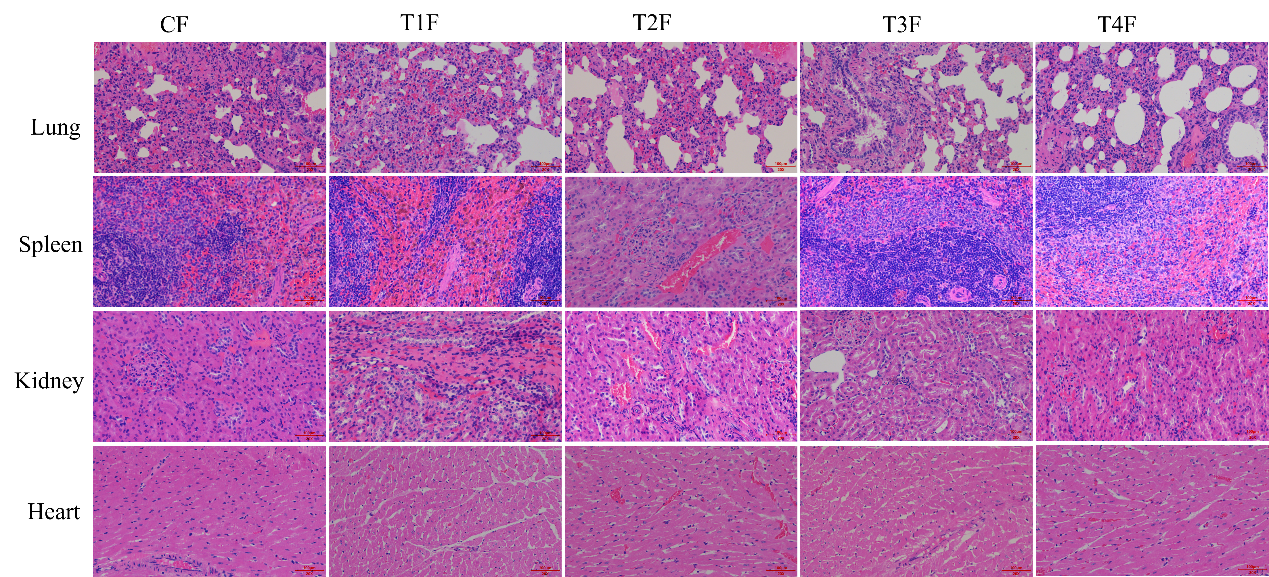


S5 The Hematoxylin-eosin staining of livers tissues from female rats


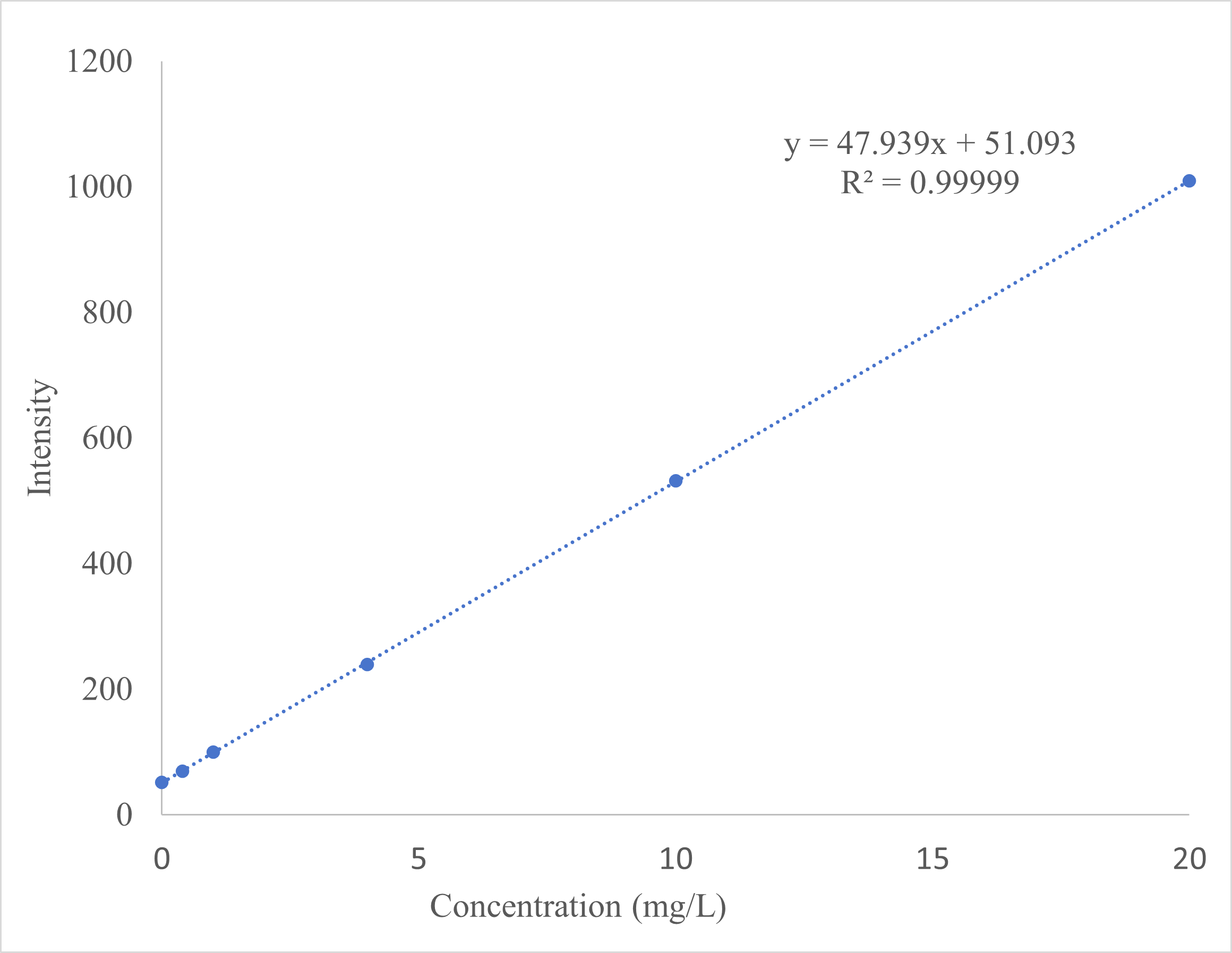


S6 Standard curve of selenium.

The analysis was performed in compliance with the Chinese national standard GB/T 13883-2023. Briefly, 0.5 g of each feed sample was accurately weighed into a digestion vessel, followed by the addition of 8 mL of ultrapure nitric acid and 10 min of room-temperature soaking for pre-wetting. After supplementing with 2 mL H_2_O_2_, the mixture was settled for at least 2 h to ensure adequate pre-reaction. Microwave digestion was carried out with a three-step temperature program: ramping to 80℃ within 5 min (5 min hold), then to 110℃ within 5 min (5 min hold), and finally to 180℃ within 5 min (25 min hold). Upon natural cooling to room temperature, the digested solutions were heated at 150℃ on a to evaporate excess acid until the volume was reduced to approximately 2mL. The resulting solutions were analyzed via inductively coupled plasma optical emission spectrometer (ICPE; Agilent Technologies, USA). A series of standard working solutions (0, 0.4, 1, 4, 10, and 20 mg/L) were prepared for calibration curve construction, and selenium content in samples were quantified using the regression equation derived from the curve. The concentration of selenium in the food was determined as 104 mg/kg and the 3dupplcated samples were tested and the concentration of selenium was represented as the mean of tested values of the samples

**S7 Histopathological Scoring Rubric**

A detailed histopathological scoring rubric was established to standardize the morphological evaluation of liver and spleen tissues from rats treated with L-SeMC, based on H&E staining results. The scoring was performed by two independent pathologists in a double-blinded manner, and the average score was used for subsequent analysis. The scoring criteria are specifically defined as follows:

**1. Liver Tissue Scoring Criteria (0-2 points)**

The scoring is mainly based on the degree of hepatic interstitial expansion, as no inflammation, fibrosis, or other pathological alterations were observed in all groups.

0 points: Normal hepatic tissue morphology; hepatic lobules with clear boundaries, intact hepatocyte structure, and no interstitial expansion.

**1 point**: Mild hepatic interstitial expansion; the interstitial space is slightly widened, and hepatocyte integrity is not affected.

**2 points**: Obvious hepatic interstitial expansion; the interstitial space is significantly widened, corresponding to the macroscopic erosive changes observed in female rats of the T4 group, without hepatocellular damage or other pathological signs.

**2. Spleen Tissue Scoring Criteria (0-2 points)**

The scoring is mainly based on the degree of splenic enlargement and structural integrity, as no inflammation, fibrosis, or other pathological alterations were observed in all groups.

0 points: Normal splenic tissue morphology; intact splenic parenchyma, regular arrangement of sinusoids and cellular components, and no splenic enlargement.

**1 point**: Mild splenic enlargement; slight increase in splenic volume, with intact splenic parenchyma structure.

**2 points**: Obvious splenic enlargement; significant increase in splenic volume (consistent with the average spleen weight of 1.11 in female rats of the T4 group), with intact splenic parenchyma structure and no structural damage.

**3. Additional Notes**

All experimental groups showed no signs of inflammation (e.g., inflammatory cell infiltration), fibrosis, cytoplasmic vacuolization, or cell necrosis in both liver and spleen tissues. Therefore, the scores for inflammation and fibrosis were uniformly 0 points for all groups, and these two indicators were not included in the formal scoring criteria.
